# The feasibility of integrating an alcohol screening clinical decision support tool into primary care clinical software: a review and Australian key stakeholder study

**DOI:** 10.1186/s12875-024-02662-4

**Published:** 2024-12-02

**Authors:** Rachel Canaway, Libby Dai, Christine Hallinan, Cassandra Caddy, Kelsey Hegarty, Douglas Boyle

**Affiliations:** 1 Department of General Practice and Primary Care, The University of Melbourne, Victoria, 3010 Australia; 2 Melbourne School of Population and Global Health, The University of Melbourne, Victoria, 3010 Australia; 3 Royal Women’s Hospital, Parkville, Victoria 3052 Australia

**Keywords:** Alcohol use, Antenatal care, Australia, Clinical decision support systems, Feasibility studies, General practice, Harm reduction, Preconception care, Pregnancy, Software tools

## Abstract

**Background:**

This study explored the feasibility of integrating a clinical decision support tool into general practice clinical management software in Australia to prompt for alcohol screening among patients who are pregnant or planning a pregnancy. The study aimed to increase understanding of what is an appropriate and acceptable clinical decision support tool, the circumstances when a prompt to use such a screening tool should occur, and the barriers and enablers of successful implementation.

**Methods:**

This feasibility study employed a mixed methods approach and purposive sampling to identify key stakeholders to interview. Participants included vendors of clinical software used in Australian general practice, clinicians in general practice, and relevant others. Data from a literature review and 23 interviews were analysed leading to recommendations which were ‘sense-tested’ by an additional 22 stakeholders.

**Results:**

Although there are at least 18 clinical software packages used in Australian general practice, it is feasible to integrate an alcohol screening tool for pregnancy into software for the majority of general practices in Australia. The AUDIT-C alcohol screening tool for pregnancy was widely accepted as suitable for such a purpose. Clinicians suggested the greatest barriers to screening were lack of time within antenatal consultations and insufficient remuneration for longer consultations. Many clinicians saw opportunity for introducing a multifunctional antenatal tool that could incorporate screening and clinical decision support for alcohol, tobacco and other substance use, mental health, domestic and family violence and potentially other areas informing healthy pregnancy. It could also be used opportunistically for preconception screening and counselling. Deployment of the tool could be supported by an education campaign from professional associations.

**Conclusion:**

The integration of a tool for screening for alcohol use among women who are pregnant or planning pregnancy into general practice clinical software is feasible; however, a multifunctional antenatal screening tool, incorporating other psychosocial elements, was considered more useful than a stand-alone alcohol screening tool. Codesign is needed with vendors and end-users to develop an acceptable tool that can be widely implemented. Issues with GP renumeration need also to be addressed to encourage alcohol screening pre-pregnancy and in the early months of pregnancy.

**Supplementary Information:**

The online version contains supplementary material available at 10.1186/s12875-024-02662-4.

## Background

It is well established that alcohol used during pregnancy can cross the placenta and cause significant harm to the developing baby including lower birth weight, increased risk of miscarriage, premature birth and Foetal Alcohol Spectrum Disorder (FASD) [1–3]. FASD is characterised by impaired neurodevelopmental, congenital anomalies and poor growth; it is a leading cause of preventable birth defects and disability, and can have ‘profound’ social and economic effects [1]. Rates of harm related to prenatal alcohol exposure in Australia is high [4–6]. Alcohol consumption among women, including during pregnancy, is a behaviour that spans demographics, including ethnicity, socioeconomic status, and education levels. In Australia, 55% of pregnant respondents to the 2019 *National Drug Strategy Household Survey* reported consuming alcohol before they were aware of their pregnancy, and 14.5% continued to drink alcohol after they knew they were pregnant, down from 25% in 2016 [5]. A systematic review estimated prevalence of prenatal alcohol exposure among Australian women to be 48% [7]. The strongest predictive factors for alcohol use during pregnancy in Australia are alcohol consumption prior to pregnancy and increasing maternal age [8].

To reduce harm caused by alcohol consumption during pregnancy, both Australian and international primary and obstetric care guidelines are in broad consensus that women who are pregnant or planning pregnancy should be advised by their care providers of the risks of prenatal alcohol exposure and that the safest option is not to drink during pregnancy [6, 9–19]. When clinicians selectively conduct antenatal alcohol screening, they tend to under-screen those who are the most likely to drink alcohol [8, 20, 21] by assuming that older, more affluent and well-educated women are less likely to consume alcohol during pregnancy, whereas research suggests the opposite can be true [22–25].

Pregnancy care in Australia can be through the public or private healthcare systems, it can be claimed and the general practitioner (GP, i.e. family physician), midwife or obstetrician-led, in primary, community or hospital settings [26]. GPs are gatekeepers to specialist healthcare [27] so are normally the initial point of contact for antenatal care, which may result in referral or ongoing ‘shared care’ arrangements with hospitals. At the first antenatal visit the GP can confirm the pregnancy, organise tests and ultrasound, review existing medications, discuss diet, exercise, smoking, alcohol and care options, and provide referrals [28]. Most Australian women have seven to ten antenatal appointments and two ultrasounds, the first at 11–13 weeks and the second at 18–20 weeks [26]. While primary care professionals often have established therapeutic relationships with their patients, consultations are typically financed for 10–15 min, so timing and context of discussions around alcohol use during or prior to pregnancy presents challenges when there are competing priorities [29, 30]. GP appointments attract Medicare Benefit Scheme (MBS) rebates.

Medicare is Australia’s publicly funded universal health insurance scheme that provides fee-for-service payments to GPs and other recognised clinicians [31]. Reason for visit directs the item number that can be claim and therefore the fee claimable by the clinician. Usually, the smaller the payment to the GP, the shorter will be the consultation. Due to a longstanding ‘freeze’ on MBS rebates, it is now common for GPs to charge a ‘gap’ payment to compensate for their time [32]. Some Medicare item numbers, such as item 16,591 which provides remuneration specifically for antenatal alcohol screening as part of a psychosocial screen, can only be claimed at 28-weeks of gestation and only once per pregnancy. Antenatal consultations (Medicare item number 16500) are capped at a lower rate than a long consultation for a non-pregnant patient.

Australian research among primary healthcare providers outlines behaviours and barriers preventing effective intervention by healthcare professionals around alcohol use among women who are pregnant or planning pregnancy [30, 33]. To address this, primary care clinicians have suggested that if relevant prompts to screen for preconception or antenatal alcohol use were included in their clinical software systems, i.e., as clinical decision support systems (CDSS), then consistent screening in primary care would be more likely [30]. However, there are at least 18 clinical software packages used in general practice in Australia (see Supplement I) and it may not be feasible to integrate prompts to screen for antenatal alcohol use into all of them. Outside of the two most used clinical software systems, BP Premier (vendor, Best Practice) and MD Clinical (vendor, MedicalDirector / Telstra Health), the 16 smaller players including Zedmed, Meditech and MediRecords, “collectively have somewhere between 12 and 15% of the (GP) market share” [34]. In Aboriginal Community Controlled Healthcare Organisations, Communicare (vendor, Telstra Health) has the majority share (Supplement I).

CDSS interact with clinical software to support patient management for a large number of conditions [35–37]. Their functionality can include computerised alerts, recalls and reminders; delivery of pertinent clinical guidelines, patient data reports, documentation templates, clinical workflow tools; and prescribing guidance [36, 37]. Differences in their technical and clinical interface functionality provide options to deliver passive prompts, enabled by the end-user to initiate and generate clinical advice, or automated prompts that do not require end-user interaction to proceed, and active prompts, where the prompts are initiated automatically by the system and must be interacted with to proceed [38].

### Screening tools for alcohol use during pregnancy

Many different tools are used to screen for prenatal alcohol exposure in a variety of Australian and international health care settings (Supplement II). The most familiar tool in the Australian general practice setting is the Alcohol Use Disorder Identification Test (AUDIT) [39] and its derivatives the AUDIT-C (AUDIT-consumption subset) and the AUDIT-C for pregnancy [40–42].

Our review (see Supplement II, III) highlighted four features of a prenatal alcohol screening tool that make it appropriate for the Australian general practice context: sufficiently brief to be used in a typical (short) GP consultation; sufficiently sensitive to detect low or infrequent levels of alcohol use, including ‘special occasion’ drinking (which is drinking on special occasions (or difficult times)’ outside of a ‘usual’ pattern [43]); acceptable to primary care clinicians and patients alike; and validated in an Australian clinical practice context**.**

Of the tools identified, AUDIT-C for pregnancy [44] best fulfilled the Australian general practice use criteria because it is speedy to administer, already widely used and is validated in the Australian general practice context including in Aboriginal health settings [41, 45, 46]; but notwithstanding the critique that its use of ‘standard drinks’ requires careful explanation to patients, and that there may be a need for an additional question to capture ‘special occasion’ drinking. Other tools having potential value in the Australian setting are the Grog Survey App [46–48], ASSIST [49] and IRIS [50, 51], although none are without limitations.

### Study rationale

Australia’s Foundation for Alcohol Research and Education (FARE) commissioned this study as prompted by earlier research among primary care providers that found: “lack of cues and requirements create an opportunity deficit to discuss alcohol in pregnancy” [30]. The study reported below was commissioned specifically to determine the feasibility of integrating a decision support tool for alcohol screening into general practice clinical software, in acknowledgement that most pregnant women initial visit a GP and that clinical software used in general practice is different to that used in other health sectors. FARE proposed eight guiding research questions, four overarching aims and research components including: review of relevant literature, clinical software used in Australian general practice, and clinical decision support tools for alcohol use management; and consultation with software vendor and primary care stakeholders. The research aims were, first, to identify an appropriate and acceptable clinical decision support tool for alcohol screening of patients who are pregnant or planning pregnancy to be used by healthcare providers in primary healthcare settings in Australia, and the circumstances when a prompt would occur. Second, to determine stakeholders’ perceived benefits, barriers and enablers of implementation of a clinical decision support tool into general practice clinical software for alcohol screening during pregnancy. Third, to identify the most appropriate clinical software vendor(s) to target for implementation. Lastly, to review the process, cost and timeframe for development and implementation of an integrated screening tool into general practice clinical software systems.

To succinctly convey the outcomes of this broad study, we focus here on results from the first two aims, guided by the following questions posed by the funder: What are the benefits of integrating a prompt into general practice management software for alcohol screening? What is an appropriate patient criteria to initiate a prompt for alcohol screening for women who are pregnant or planning pregnancy? What alcohol screening tool would be acceptable to health professionals as a prompt? What are the barriers and enablers to successful implementation? See Supplementary Material, including a summary of clinical software systems used in Australian general practice (Supplement I), a summary of alcohol use screening tools for health care settings (Supplement II) and the review methods (Supplement III).

## Methods

### Study design

This multi-phased, mixed methods study used a Health Policy and Systems Research (HPSR) approach which draws on social science perspectives and uses a problem rather than method-driven approach to develop ‘real-world’, feasible solutions to support the development and implementation of applied policy and health system change [52, 53]. The study was undertaken between November 2022 and May 2023. The research team included two academic GPs, a health data informatician, and two health policy/systems researchers [one a health social scientist, the other a biostatistician / informatician (both with clinical backgrounds]), and a public health PhD candidate. Ethics committee approval and informed consent from participants were obtained (see Declarations). The funder did not play a role in the research design, data collection, analysis, interpretation, report or manuscript writing.

A rapid literature review was undertaken to establish base knowledge (see Supplements I-III and [37]). The review informed development of a prototype screening tool (developed by author LD) (Supplement IV), the interview schedule for primary data collection (Supplement V), and provided context for data analysis and development of recommendations. The prototype tool was used as a discussion prompt during stakeholder interviews. We gathered input first from stakeholders via semi-structured interviews and second using a questionnaire to test acceptability of the draft recommendations that resulted from the first phase of data collection (Supplement VI). Interviewees were invited to review the draft recommendations that resulted from their interview responses.

### Recruitment and sampling

For the interviews we used purposive sampling to target key stakeholder representatives from vendors / creators of clinical software packages used in Australian general practice (hereafter referred to as vendors), GPs and practice nurses (including from Aboriginal Medical Services), representatives from professional associations representing primary health care providers (hereafter referred to as professional association representatives), and relevant researchers. Representatives from the Royal Australian College of General Practitioners (RACGP) Specific Interest Group in Antenatal and Postnatal Care, the Expert Committee–Practice Technology and Management were specifically approached. Information about the study with a QR code linking to a recruitment information page was also included on a poster presentation at the RACGP GP22 conference in November 2022. GP and primary care nurse participants were also recruited including through snowball sampling.

To recruit clinical software vendors, the list of vendors (Supplement II) was used as an invitation register. Invitations were sent to 16 of the 18 vendor organisations (those that had public contact details) via email or web-based ‘Contact Us’ pages. A follow-up email or phone call was used to prompt non-responders.

For the ‘sense-testing’ of the recommendations, an invitation with questionnaire link was emailed to all interviewees and all vendors initially contacted (including those who declined to be interviewed), and in addition, widely distributed via the RACGP Research Noticeboard (publicly accessible), GPs Downunder Facebook page (a closed Facebook community of around 5,800 GPs in Australia and New Zealand), the Victorian primary care practice-based Research and Education Network (964 emails opened by 447 recipients—www.gp.unimelb.edu.au/vicren). One reminder email was sent to those whom we had directly emailed the questionnaire link.

### Data collection

Guided by the research questions, two interview question guides were created, one for vendors, the second for all other interviewees (see Supplement V). Interview questions and screenshots of the mock software-embedded alcohol screening prototype were circulated to potential participants prior to the interviews. The prototype tool incorporated an adapted Alcohol Use Disorder Identification Test (AUDIT)-C (consumption subset) for pregnancy screening tool [37]. 

Online interviews were conducted between February and April 2023 using Microsoft Teams. All interviews (*n* = 23) were audio-recorded, 22 were video recorded, and all were transcribed verbatim. Interviews with 10 GPs and two practice nurses were undertaken by LD, the other 11 were conducted by RC and CC. Interview length ranged from 19 to 62 min (average 44 min). Because of the high number of vendors, and to include healthcare professionals from different jurisdictions, we increased the number of interviews from the small number suggested by the funder.

After initial analysis of interview data, we developed a draft set of overarching recommendations which we ‘sense-tested’ among stakeholders using an online questionnaire which was open for anonymous feedback from 27th April to 22nd May 2023 (Supplement VI). Feedback during piloting urged that we shorten the questionnaire by removing most demographic questions or anything potentially identifiable, including whether the respondent had been an interviewee. The questionnaire was hosted on the University of Melbourne’s instance of the Qualtrics platform [54]. Respondents were asked to rate their agreement with the draft recommendations via a five-point Likert scale (Strongly Agree, Agree, Neutral, Disagree, Strongly Disagree) and optional long answer comments.

### Data analysis and reporting

Interview transcripts were uploaded into QSR NVivo 12 Plus [55] and thematically analysed by question to determine emergent themes [56]. Guided by the HPSR approach and informed by our literature review and emergent themes, we developed a draft set of recommendations to guide the design, development and implementation of the proposed screening tool, and other recommendations as suggested by the emergent themes.

Feedback from the draft recommendation ‘sense-testing’ was reviewed and recommendations finalised; these were reported to the funder in a confidential report. The results below present themes arising from the interviews and include feedback from the ‘sense-testing’ when relevant to further illustrate a perspective, and a high-level summary of the recommendations.

## Results

### Participant characteristics

We received expressions of interest from 21 GPs and 3 practices nurses. Of the 16 vendor organisations contacted, seven agreed to an interview, four declined and there was no response from five. A breakdown of the 23 interview participants and 22 questionnaire respondents, by stakeholder type and jurisdiction, is shown at Table 1. The questionnaire was commenced by 32 eligible people and completed by 22. A total of 54 free-text comments were left by 12 of the 22 respondents. No respondent left a comment for every recommendation. Due to the anonymity of ‘sense-tester’ questionnaire responses, we do not know which of the testers were also interviewees.

**Table 1:** Participant types and their jurisdictions

Participant type	Interview participants n (%)	‘Sense-testers’ ^a^ n (%)
General practice clinical software creator / vendor	7 (30.4)	3 (13.6)
General practitioner	10 (43.5)	15 (68.2)
Practice nurse	3 (13.0)	3 (13.6)
Representative from a primary care-related medical college or association	2 (8.7)	1 (4.6)
Relevant academic	1 (4.4)	N/A
**Total**	**23 (100)**	**22 (100)**
**Associated with Aboriginal Medical Services**	Unknown	5 (22.7)
**Jurisdiction**		
New South Wales	3 (13.0)	2 (9.1)
Northern Territory	1 (4.4)	0 (0.0)
Queensland	1 (4.4)	2 (9.1)
South Australia	0 (0.0)	0 (0.0)
Tasmania	1 (4.4)	0 (0.0)
Victoria	7 (30.4)	12 (54.5)
Western Australia	3 (13.0)	2 (9.1)
National (vendors of clinical software used in general practice)	7 (30.4)	3 (13.6)
Did not say	0 (0.0)	1 (4.6)
**Total**	23 (100)	**22 (100)**

^a^Sense-testers completed a feedback questionnaire where they reviewed the draft recommendations developed as a result of the interviews and informed by the literature review

### Participant perspectives

The results summarise the perceptions of participants on benefits of integrating a prompt for alcohol screening for pregnancy into general practice clinical software, appropriate criteria to initiate a prompt for screening, vendor perspectives on integrating a tool, clinician perspectives on an acceptable tool, barriers to implementation, and enablers of successful implementation. The section concludes with a high-level summary of the recommendations that were developed by synthesis of interview data supported by the literature review (see also Supplement VII).

### Benefits of integrating a prompt into general practice management software for alcohol screening

Most clinical software vendors saw value or benefit in integrating an alcohol screening tool specifically for routine use with pregnant patients. Benefit was described as “self-evident”, “making clinical point-of-care easier”. It was said that most GPs “appreciated… little prompts and reminders…any kind of subtle guidance.” However, one vendor was less sure:

I guess the benefit would be you would be directing clinicians to specific information to share with patients … but are you enhancing what the clinicians do around alcohol screening in pregnancy? … Do you think, do you honestly believe that pregnant women or women that want to get pregnant aren't getting enough advice about alcohol? … Is that true or not? I don’t know. (Vendor 5)

Not all interviewed GPs routinely screened for alcohol use among pregnant patients; however, the majority saw value in having a standardised screening tool and prompt as it could potentially enhance rates and consistency of screening by GPs, especially for doctors, who have not yet “bedded down” how to routinely ask patients about alcohol use:

Because I've bedded it down so well (asking pregnant women about alcohol use) … I don't need your decision support... But (it would be helpful) for my colleagues who haven't bedded it down, and I would say that's the majority. (GP09)

It was observed that when informal screening takes place without standardisation of documentation–which a screen prompt should accommodate–the value of the collected information in limited as future clinicians will not be able to easily access past screening outcomes “because you’re not going to read through everybody's notes every single time” (GP03).

The language within the prototype tool (Supplement IV), and guidance provided by the tool on management depending on screening results, was thought to allow inexperienced and trainee clinicians to feel more confident to conduct screening and “support the use of non-judgemental language”. Having the screening tool embedded, with the expectation that it be used with all pregnant patients, also removed the sense of passing judgement when GPs or nurses could assure patients that they are “routine” questions asked of everyone. An automated visual prompt could also act as a facilitator for initiating challenging conversations with patients:

(By using the formal screening tool) I can be the intermediary or the interface. I can say to patients: ‘Look, this prompt wants me to do this.’ ... Then they don’t feel that it’s necessarily coming from me, who is more their ally and their advocate …. I find it helpful if something pops up and I can go: ‘Look, these are the recommendations. How do you feel about that?’ … I find patients really respond to that sort of informal approach that I can have while engaging with a more formal resource. (GP08)

### GPs views on appropriate criteria to initiate a prompt for screening

GP participants considered that entering a diagnostic code for a current pregnancy was the only feasible criteria for initiating an automatic prompt for alcohol screening in general practice software. There was consensus among clinician participants that every patient known to be pregnant should be asked about alcohol use: “I think you shouldn’t make any assumptions about anything. You should just ask everybody” (GP academic).

To guide discussions during pregnancy planning and preconception care, interviewed clinicians wanted the tool accessible outside of the automated prompt (visible on the clinical desktop / interface, e.g., drop down menu). They considered that the time constraints of general practice consultations and the rarity of patients proactively seeking preconception care, made the inclusion of an automated prompt triggered for pregnancy planning less feasible.

I don’t see many people for prenatal counselling to be fair... I'll be seeing people once they're already pregnant. The only other time I see them prenatally is if they're having trouble conceiving. (GP05)

GP participants were not aware of a diagnostic code or template in general practice clinical software systems to capture ‘Planning Pregnancy’, so first the field would need to be included in the software; however, they highlighted that preconception advice tended to be delivered opportunistically in the context of other consultations rather than in a standalone consultation. They identified a range of time points at which they might conduct opportunistic preconception care discussions (e.g. attendances for cervical screening tests and contraceptive advice), at which point they might manually access the pregnancy-related alcohol screening tool. Not all considered that a prompt would be helpful in these contexts, as they tended to judge on a case-by-case basis whether they had time to raise the discussion and to judge how an unprompted conversation about reproductive health might be received by the patient. Repeated prompts, for example, towards the middle and end of a pregnancy, were suggested to guard against assumption that alcohol use status pre-pregnancy or in early pregnancy has not changed.

### Vendor perspectives on embedding an alcohol screening tool in clinical software

All vendors reported that their clinical software systems incorporated a mechanism to document alcohol use, however, only some included formal screening tools to structure information gathering and risk assessment about alcohol use. None of the vendor interviewees were aware of having alcohol screening tools that were specific for pregnancy incorporated into their software.

AUDIT-C was the tool most incorporated into existing clinical software packages. Its inclusion was reported by some vendors to be relatively new and did not have prompts to use it: “(the user) basically has to go to the menu to launch it themselves”; i.e. it was not set up as a CDSS that would be automatically triggered with input of patient data. Some vendors described other ways to capture data about alcohol, tobacco and drug use or history, e.g. “a couple of text fields and checkboxes”. One vendor described that within their pregnancy module, alcohol screening is one of several items present on a checklist for clinicians, i.e. they provided a place to document that an alcohol screen had been conducted, but no mechanism to conduct that screen.

Vendor participants, mostly, did not have a way to know how often alcohol screening was undertaken by software users–in particular, among those presenting as pregnant: “being on-premises (loaded onto the user’s computer or server as opposed to cloud-based software) and having legacy architecture (software system based on dated technology), we don’t get a lot of data about the tools that are used… I couldn’t give you any accurate understanding on how often it (AUDIT-C) is actually used” (vendor 3). The exception was with the cloud-based software systems where, without access to patient specific information, the vendor could generate an audit (count) of how many times alcohol screening had been interacted with. One cloud-based vendor had a “consented sample” of practices from which they could, for example, check how many times the AUDIT-C assessment was used, which fields were completed and if done during antenatal or postnatal consultations (if pregnancy was recorded or if pregnancy or postnatal care was listed as a reason for visit).

### Clinicians’ perspectives on an acceptable screening tool

Clinician participants suggested that the features outlined in Table 2 should be incorporated into an alcohol screening tool incorporated into their clinical software. These included risk stratification scores charted over time and auto-population of fields, resulting in the provision of ‘high-level’ management advice and one or two resources that are unlikely to be quickly outdated. The tool should be ‘optimised’ for a variety of communities and settings, including Aboriginal and Torres Strait Islander communities.

**Table 2:** Features of an acceptable alcohol screening tool for general practice, clinicians’ perspectives

Suggestion	Feature description or justification	Participants’ comments
**Automated documentation of risk stratified scores**	- Helpful to generate scores to risk stratify patients - Risk scores need to be automatically and appropriately documented o So other clinicians can find them o Because if it’s not automatic, it probably won’t be adequately recorded	“We're all prompted to put a record into My Health Record. Do we all do it? No, because we don't have time. We try our best… but I don't think we hit the targets for doing the uploads. It would make far more sense to have it (alcohol risk scores) automatically uploading… practising defensible medicine and being able to document everything is vitally important. Have a prompt that is going to do a lot of the work of recording as well.” (GP03)
**No overwriting of scores**	- Allow assessment of a patient's alcohol risk profile across different timepoints to see change over time for reflecting on management progress	“The problem with smoking assessment too (as well as recording of alcohol consumption), is that it doesn't record previous consumption or use. You can only put in what they do now and when you update it the previous is gone and … if they used to be a very heavy drinker, but now they drink very rarely or not at all. That is not captured very well in the data.” (GP College rep)
**Field auto-population**	- Auto-populate relevant fields in health summaries, electronic referrals and shared maternity care records (with patient consent) and thereby streamline workflow to avoid duplication of effort	“So if it auto-populates when we need to do a referral that is very helpful because it just saves you time.” (GP02) “I think what’s really helpful, if it could automatically link into the referral, for instance. Click on the link and up comes your template for the referral, that would be great.” (GP03)
**Data accessible for reporting**	- Having reports of the captured data readily accessible to support quality improvement activities (noting that quality improvement activities are necessary to maintain accreditation, and some practices also required this to maintain funding)	“Because of our CPD that we have to do “Measuring Outcomes”; isn’t that going to be so much easier if you can do a nice search for your scores? An audit for someone could be: In the last thirty antenatal cases that you saw, how many did you do drug and alcohol screening? And it's automatically there, with your tool. (GP03)
**Provide advice that is high level and non-binding**	- Advice provided by the tool should not include specific recommendations that may not be feasibly actioned, or referral pathways that are not locally available - Management for individual patients is best decided by clinicians who know their patients and the availability of local services, not by the tool	“We have one psychologist, and for adults it's over 12 months wait. And a psychiatrist its 18 months, so having a pop up that says “Refer to psychiatrist” is actually not helpful at all. What you do with it I guess is still up to the clinician.” (GP04) “You’re not going to be able to make resources for every single clinical context because you don’t know who is sitting in the room with the doctor and you don’t know what the resources are that the doctor and the patient have at their disposal.” (GP10)
**Include only a few key resources**	- Include one or two contacts for peak organisations - Unfeasible to maintain local referral resources given the constantly changing landscape of services with funding cycles	“It would be very hard I guess for you guys to roll out a thing that's localised. Like for me, if I need mental health support, I'd be calling our local mental health triage number, but that's very different to what you would do if you were in (another city), for example.” (GP04)
**Suitable for a range of communities**	- Ensure the tool is optimised for a range of communities, including regional and remote, not just metropolitan areas of the “southern states”	“If you were talking about rolling the software out to places that have (Aboriginal) communities, then I would do a big consultation process. Get a council of Elders together to ask them whether they think it's appropriate to ask these questions. And of course, what you might ask in Sydney is going to be different to the Northern Territory.” (GP06)
**Accessible outside of automated prompting**	- There should be more than one way to access the tool. The tool should be readily accessible on the computer desktop or easily located in the clinical software, for opportunistic screening such as when a patient is planning pregnancy	“That should be a regular thing that we should be screening, even before getting pregnant, of course. But if not, then reassessing again when someone is pregnant.” (GP02)
**Passive prompts**	- Avoid ‘alert fatigue’ due to forced interaction with prompt. Prompts should not be interruptive of workflow	“Some people don't even look at the prompts and just put, you know, cancel, cancel, cancel.” (GP02) “It would be a barrier if you couldn’t move onto the next page (of the tool) without filling it in.” (GP)
**Repeated prompts**	- Repeat prompts, for example, towards the middle and end of a pregnancy, could be useful to guard against assumption that alcohol use status pre-pregnancy or in early pregnancy has not changed - When care is shared between general practice and antenatal clinics, prompts could be scheduled to fit the visit schedule	“I think it's a good reminder, when you put in your antenatal check, but it would be really good to have the option of using it… prenatally or even in the middle of a pregnancy or postnatally as well.” (GP01) “If we are doing shared care, then we have set times when we see them with their local antenatal clinic. So that's when we would do them (screening).” (GP02)
**Multifunctional rather than single purpose**	- A large volume of risk relevant information needs to be gathered in an early antenatal consult, such that some questioned the sense of integrating a tool just for alcohol screening - It was suggested that an antenatal clinical decision support tool could, as a minimum, include screening for alcohol, nicotine and other substance use, but might also include elements of a psychosocial screen, including mental health, domestic and family violence, nutrition, housing - Framing discussion about alcohol use within a broader assessment framework could be appealing to some clinicians	“The decision support on alcohol intake is but one part of a whole bigger issue that needs to be dealt with.” (GP College rep) “If we add nutrition into that as well, you ask are they taking folic acid, tick the box, you've asked about diet, tick the box, you asked are they working, tick the box. Having everything built into that would be great.” (GP03) “It doesn't make sense to me… to have a tool that only asks about alcohol … It's taking one thing when screening for domestic violence is important, smoking is important, not eating soft cheese that might give your baby Listeria is important. So it doesn't work from a practical point of view, having alcohol by itself. (GP academic) “You need to include other drugs and when we're talking psychosocial, to me, it's housing, it's food, it's violence, it's alcohol, it's smoking… I would include a bit more of the bigger picture.” (GP06)
**Multifunction with Save and Return feature**	- A multifaceted screening tool should have the functionality to partially complete, save and return, to allow clinicians to pivot the focus of their consultations in response to what a patient discloses during screening	“I wonder if rather than the tool being a whole, like you administer everything all at once, having the functionality… so you could do it over like a couple of consults. If it's just too much to do in one.” (GP01)
**If standalone: AUDIT-C for pregnancy**	- If the tool was alcohol only, then AUDIT-C for pregnancy was considered appropriate to enquire about and quantify alcohol use. The brevity of AUDIT-C for pregnancy was a strong point	“If it's only a few buttons (clicks on the screen), like the Audit C tool… So long as it's not 10 questions, then I don't think that's a problem” (PN1)

All interviewed clinicians were familiar with and/or had used the AUDIT-C screening tool, though not all had seen the adapted AUDIT-C for pregnancy [44]. Most felt that if a formal screening instrument was to be adopted, AUDIT-C for pregnancy was an appropriate screening tool, because of its brevity and specificity, to enquire about and quantify alcohol use. Despite this, there were differing opinions as to whether a formal screening tool would work in their own clinical consultations. Additionally, there were repeated suggestions for a multifunctional tool incorporating additional measures useful for screening during pregnancy, that could be partially populated, saved and returned to; for example, tobacco, other drugs, mental health, nutrition, family violence.

### Barriers to screening and screening tool implementation

The barriers to successful and efficient implementation of an alcohol screening decision support tool, whether standalone or multifunctional, embedded into general practice clinical software are summarised at Table 3 with key illustrative quotes. The greatest barrier to using formal alcohol screening tools cited by GP participants was lack of time, related to this was pregnant patients booking short consultations when a long consultation is warranted, competing priorities, and as outlined later, the structural barrier of insufficient remuneration through the Medicare system which exacerbated lack of time. Clinician participants highlighted that alcohol use is one amongst many complex and often interlinked factors that contributes to pregnancy health outcomes and there is lack of incentive to prioritise it over other factors.

**Table 3:** Barriers to alcohol screening, and use and development of an alcohol screening decision support tool embedded in general practice clinical software

Barrier	Barrier description	Participants’ comments
**Clinicians’ lack of time**	- Short consultations leading to not enough time to do everything - Competing priorities – not enough time to do everything	“The barrier to use of the tool is time mostly …. The patient has often booked a (short) appointment… The Doctor's agenda is that they have an allocated time for that appointment, which is 10–15 min, 20 min depending on the practice. And they're not going to cover most things in that short time frame… As soon as a woman walks in who says they're pregnant you've got a massive agenda ahead of you … (the doctor’s) got more people waiting and they've got to get them out of there. So they do something small, get them out.” (GP College rep) “Alcohol is only one tip of an iceberg in how to manage a pregnancy appropriately… there’s so much information that it’s very hard to touch on the important bits and know what to deal with.” (GP College rep)
**Clinicians’ lack of incentive**	- Lack of incentive to prioritise alcohol screening over other aspects of an antenatal consultation	“If things aren't mandated then people just don't bother. I don't mean that in a bad way. I mean, it's maybe just not thought of as relevant.” (PN)
**Alert fatigue (ignoring pop up alerts)**	- There are already many ‘pop-up’ prompts in clinical software that clinicians ignore	“Some people don't like pop ups because they feel that the devalues their clinical judgement. I don't think it's such an issue with alcohol prompting, but there are obviously issues with alert fatigue that can be problematic, especially with people just switching off the system.” (Academic GP) “I can see it's important, but we've already got some prompts… but to be honest, most of the time they ignore them.” (GP06)
**Clinical judgement**	- **Deliberate choice** not to ask about alcohol use or to mark a woman as pregnant (which means a prompt to ask about alcohol use will not be triggered). o Protection of women’s’ privacy and safety as reason not to document pregnancy in the clinical software o Believing that doing so would erode trust and report - **Assumptions** about who should be screened, e.g. that patients have sufficient health literacy to know not to drink alcohol when pregnant	“So we never make her pregnant on the system because other people might come and see that she is pregnant and then tell some other people and then they'll—there's a lot of reproductive coercion here.” (GP06) “In a normal consult, if I'm building rapport, I might judge that… this patient is still learning to work with me or still starting to trust me, from the nonverbal cues. And so I might ask that next time.” (GP01) “It could be that I'm actually not picking it up (drinking alcohol when pregnant) because I'm not asking.” (GP01)
**End-users not capturing data in fields that would trigger a prompt**	- Clinicians’ lack of awareness of features of the clinical software that they use - Lack of time	“A lot of doctors don't put in that a patient is pregnant …(if they did) then the software already has decision support smarts in terms of not prescribing certain medications to pregnant women or alerting you that this is a category X drug and should be ceased or whatever.” (GP College rep) “I am aware that there is the Audit C tool on Best Practice. However, I am also aware that it is rarely used, like many screening tools on software…(because of) lack of training, lack of knowledge, lack of time.” (PN)
**Lack of time and incentive for vendors**	- Lack of funding and incentive for vendors to make time to schedule develop a new screening tool in an already full production and update release cycles	“One barrier we have, and this will probably be the same for most clinical systems, but we have a very full road map. It's very difficult to get things in there (into development and production) … our schedules are always full.” (Vendor 3) Well the first barrier would be to get it into the software, that's a huge barrier knowing the software development companies. You know, they can't, they won't develop and integrate other, much bigger issues unless they're paid, so they'd need significant funding to develop those sorts of tools. (GP College rep)
**Lack of funding to develop and integrate the tool**	- Who pays to development the tool?	Who’s going to pay for it? You know the GP software vendors, if they don't get paid and they're asked to put it in, they're going to say, ‘Well we'll early develop it if we can make some more money out of it, because it costs us money to develop the software, so who's paying us? If the government's not paying us or special interest groups not paying us to do it, the purchaser of our software is going to have to pay for it, that's the GPs.’ And the GPs are going to say “Well, I don't need that. It's not worth (paying for)’. (GP College rep)
**Insufficient consultation with end-users and developers**	- Specifications for the tool being developed with insufficient vendor or end-user consultation, leading to technical issues, problems in development and adding time to development	“It can be very difficult and adds a lot of time to the process if the specs aren't clear or ambiguous and there's a lot of back and forth that happens and what should have taken two weeks development ends up taking two months because you got halfway and something was broken, and you have to go back and fix it.” (Vendor 3)

Another significant barrier raised by both vendors and clinicians, was ‘alert fatigue’ caused by high numbers of pop-up alerts associated with existing embedded screening or warning tools. Some vendors had policies where they actively limited the number of automated prompts (pop-ups) that a GP would receive. Vendor participants knew that many GPs actively disabled pop-up alerts, even when they were alerts for items requested by end-users. GP participants highlighted that when they experience high volumes of prompts, that each individual alert carries less weight, and leads to desensitisation to alerts even where they may be clinically relevant. GPs indicated this barrier was less of a concern when prompts were ‘passive’ or non-interruptive to workflow, i.e. that they would be visible on the screen without requiring the clinician to interact with them to continue their consultation.

Clinical judgement was cited as another reason why screening of pregnant women for alcohol use may not occur. This could be a deliberate choice or driven by assumptions. One participant GP described reading cues from patients and making judgements on whether a formal screening tool would detract from rapport-building and potentially whether the patient would return. Another GP reported that their patients had such high health literacy that alcohol use “isn’t an issue”, but then reflected: “Or it could be that I'm actually not picking up (alcohol use) because I'm not asking”. Clinical judgement was also cited as a reason why a formal tool for alcohol screening might not be used. This was illustrated in an Aboriginal community setting in the Northern Territory, where the use of a formal tool detracted from building rapport and gaining trust. This did not mean that information about alcohol use was not sought:

Obviously, a lot of Aboriginal population, it's all verbal… So you talk the story… you use a narrative to tease it out rather than a tick box thing. So I don't write notes - well, I do at the end, but I don't sit there and go da, da, da [imitates typing]. (GP06)

The issue of the software end-users not knowing how to fully utilise the features of their clinical software, and for example, not marking a patient as pregnant (using a diagnostic code) within the clinical software (EMR), was raised. Failing to do so may mean automated alerts related to alcohol screening would not trigger.

The absence of a suitable trigger for a screening prompt before conception was considered a tricky barrier for incorporation of a pre-conception screening prompt into the software. One vendor described this as a problem that could be overcome “as long as you’ve got business logic, you understand what you’re searching for and understand what the rules are and you can translate that to the system, (then) you can do that.”

Vendor participants also raised lack of time associated with potential unwillingness to make development of a new screening tool a priority in their already full production and update release cycle schedules. Another barrier to development of an integrated screening tool cited by vendors, was insufficient consultation on requirements and therefore lack of clear specifications at the outset thus requiring much “back and forth” between client and vendor–adding to the problem of lack of time.

### Lack of appropriate resourcing, a structural barrier

As flagged above, lack of time to screen for alcohol use during pregnancy was considered to be, in part, driven by limitations in Medicare’s fee-for-service funding model for antenatal care. GP participants described the negative impact of the capped value of an antenatal consultation (Medicare item number 16500) lower than the long consultation that can be charged for a non-pregnant patient. This meant that if delivering comprehensive pregnancy care–which takes time–GPs were forced to decide between accepting a financial penalty or passing the cost of taking additional time onto patients in higher out-of-pocket fees to cover the gap.

Most GPs are using time-based item numbers… We're only supposed to use item number 16500 for all antenatal visits, that has a rebate of $42.40. If I spend 30 minutes with a woman, that's a $135 private fee. She's getting $42.40 back. You take me to another 15 minutes and now she's being charged $175 … but she's still only entitled to a $42.40 rebate… I think the conversations need to change about how long (Medicare) allow for the consultations that we have, and we need to build in enough time to do the job properly. (GP9)

Medicare requires you to bill the most appropriate consultation item number, and yet if you're not qualified and haven't done the training to do pregnancy counselling, you can't bill that item number, you can only bill a standard consultation item number. And so that means that you're also limited in how long you can spend with a patient. (GP College rep)

A further structural disincentive to appropriate screening was that the Medicare item number that specifically remunerates antenatal alcohol screening (Medicare item 16,591) as a component of a psychosocial screen can only be claimed after 28-weeks gestation and only once per pregnancy and pointed out that this does not align with clinical practice guidelines that state alcohol screening should be conducted early and often for every pregnancy. GPs reported that funding disincentives did not change their commitment to delivering high quality antenatal care but expressed frustration that this care was devalued or made less accessible.

GP participants working in locations spanning major metropolitan settings through to remote areas of Australia indicated that they struggled to access specialist care for their patients even when clinically indicated. One GP working in an under-resourced remote community where rates of problem alcohol use were very high described a sense of futility, that gathering data about alcohol use through formal screening is little more than “just ticking boxes” unless there is the resourcing to address the underlying drivers and manage the impacts of alcohol use.

### Enablers

Interviewees suggested ways to support both the development of an alcohol screening tool integrated into general practice clinical software and increasing rates of alcohol screening among pregnant women in general practice. To develop the tool, funding, consultation and co-design; to use the tool in practice, education and training; to increase screening rates in the face of time constraints, use of pre-consultation questionnaires was suggested, and also reform of the Medicare funding system that was described to push GPs to short antenatal consultations.

For developing the tool, vendors wanted funding and well thought through specifications *before* being approached to carry out development work, but some wanted early consultation on tool specification development to ensure “alignment and agreement from industry” and technical feasibility. To ensure the tool is practical and enhances clinician workflow rather than adding to workload, clinicians wanted to be involved in its co-design and ensure sufficient piloting and testing of the included messaging.

GP and PN participants suggested education and training of end-users accompanying the release of the software to be a key facilitator to its use. The training should include evidence on why and how using such a tool would make a difference in terms of clinical outcomes for patients, as well as demonstrable improvement in ease of workflow in gathering, documenting, and using health data. GPs emphasised that training usually focused on clinical aspects while technical aspects of using software systems is assumed and as such, there are many clinicians within the primary care workforce with poor digital literacy.

I think training would be important… I think you might have to have sort of two trainings. So there might be GPs that use technology really easily, so it's really just about the tools and the functionality… just a quick sort of up-skilling or education… I think there's the ones (GPs) that like really struggle with any new changes or technology, and they're going to be the harder ones. (GP01)

Pre-consultation questionnaires to screen for alcohol use (and potentially other substances and mental health, etc.) were described as an alternative or adjunct to in-consultation screening as a strategy to maximise the value of time-constrained consultations. Some GP participants reported using pre-consultation questionnaires for mental health screening in their current practice, and those who were using systems for doing this flagged the value of this in ensuring consultation time can be used as efficiently as possible. “That way we (GPs) can focus on dealing with any issues (arising from the screening) rather than trying to identify them and deal with them in the consultation.” (GP researcher) It was suggested that a ‘third party’ organisation external to the software vendor might develop the screening tool to sit outside of the clinical software, but to, ideally, integrate collated information within the clinical EMR. A barrier to conducting pre-consultation questionnaires was that it requires a mechanism for the patient to record the reason for their visit when they book, to alert the practice to send the relevant questionnaire, and few GPs reported this mechanism in place in their current practice.

To encourage longer antenatal consultations, GPs suggested that “the government (needs) to change the Medicare rules, that will make a difference. What gets funded gets done… (we need to) get permission to use time-based item numbers” (GP09). Reform of Medicare funding, to ensure appropriate rebates for longer antenatal consultations, was considered a major enabler to allow time for routine alcohol screening during pregnancy. GP participants stressed that funding must provide appropriate resourcing not simply to conduct the screening, but to adequately manage the care needs of the patient if they do disclose a level of alcohol use that requires further treatment. The issue of misalignment of Medicare funding and clinical practice guidelines, where the current funding model does not allow for alcohol screening early and often in pregnancy.

### The recommendations

The recommendations in Table 4 were a result of our literature review and stakeholder interview findings, and were a key output of the project to guide the funder in next steps for the design, development and integration of an antenatal screening tool into existing clinical software systems. The interviewees and other stakeholders were invited to review the recommendations arising. While only 32 people commenced ‘sense-testing’, the 22 who completed the questionnaire were overwhelmingly positive with one or two respondents disagreeing (4.6–9.1%) with four of the recommendations (see Table 4 and Supplement VII for greater detail). The pre-consultation questionnaire was added part way through the sense-testing process so is less tested.

**Table 4:** Agreement with screening tool design and implementation recommendations

		**Level of sense-tester (stakeholder) agreement**		
		**Agree** n (%)	**Neutral** n (%)	**Disagree** n (%)
**Screening tool design recommendations**				
A	An alcohol screening tool for women who are pregnant or planning pregnancy should be multifunctional and holistic	18 (81.8)	3 (13.6)	1 (4.6)
B	Indicating ‘Currently pregnant’ in the clinical software triggers automated prompt for alcohol screening (± psychosocial screen)	19 (86.3)	2 (9.1)	1 (4.6)
C	Pre-consultation questionnaires may be used in addition to in-consultation screening^a^	8 (80.0)	2 (20.0)	0
D	The decision support tool should be easily accessible outside of automated prompting	22 (100)	0	0
E	Generated risk scores should be informational and incorporated into relevant data fields without overwriting prior scores	18 (81.8)	3 (13.6)	1 (4.6)
F	Data collecting and collating should be streamlined to avoid duplication of work in clinical tasks and in quality improvement activities	16 (72.7)	4 (18.2)	2 (9.1)
**Development process recommendations**				
G	Clinical decision support tools should be co-designed with end users	22 (100)	0	0
H	Ensure appropriate end-user education to encourage uptake	21 (95.4)	1 (4.6)	0
I	Consultation, funding, support and clear guidance for primary care clinical software vendors	21 (95.4)	1 (4.6)	0
**Reimbursement and guideline reform to remove barriers to routine screening**				
J	Reform Medicare Benefits Scheme (MBS) rebates to facilitate antenatal and preconception screening to reflect clinical practice guidelines	20 (90.9)	2 (9.1)	0

^a^This recommendation was added late to the sense testing, hence smaller numbers of respondents

## Discussion

This feasibility study of 23 GPs, practice nurses, general practice-related professional association and clinical software vendor representatives, supports the idea of integration of an alcohol screening tool with automated prompts to use in general practice when pregnant women present. The objective of such a tool is to further reduce rates of harm related to prenatal alcohol exposure in Australia [4–6]. Another component of the study, not included in the results above but reported to the funder, was to determine the process, cost and timeframe for development and implementation of such a tool. We found integration is straightforward and feasible, and there are options in how it might be approached. Feasibility for women planning pregnancy is less straightforward as few Australian women present to their GPs specifically for preconception care, unless they are accessing assisted fertility [57, 58].

Through our review of alcohol screening tools (Supplement II and III), we determined that the AUDIT-C for pregnancy [44] met the criteria for a suitable antenatal alcohol screening tool for use in general practice settings and therefore a suitable tool for integration into clinical software. We found that software vendors and clinicians agreed that this was an acceptable tool. AUDIT-C for pregnancy overcomes the problem common to many screening tools that were developed at a time when the relationship between low levels of prenatal alcohol exposure and alcohol related harms to the foetus were less clear. As such, many other screening tools score with cut-off thresholds that have greater sensitivity for high-risk alcohol use and alcohol use disorders, but poor sensitivity for low level or infrequent alcohol consumption. With improved understanding of the impact of even very low levels of alcohol during pregnancy, many of these tools, for example, the standard AUDIT-C [45, 46], may fail to identify a significant proportion of patients who are drinking at levels that would be considered low or moderate outside the context of pregnancy. We note, however, the appetite from GPs for a broader screening instrument covering other aspects of antenatal care in addition to just alcohol screening, which would be a limitation if AUDIT-C for pregnancy was incorporated as a standalone tool.

Many of the barriers to use of an alcohol screening CDSS raised by interviewees aligned closely with those reported in Australian and international literature [37]. These included disruption to workflow within a clinical consultation due to interaction with CDSS tools, which leads to ‘alert fatigue’ or ‘prompt fatigue’ where GPs either disagree, distrust, or ignore alerts because they are delivered too many, too often [36, 59–64]. Also, negative effects on clinical autonomy and control; limitations in end-user’s IT literacy, and inadequate IT support and training challenges around content maintenance and information quality; and concern about litigation, for example, if recommendations in the tool could not be followed [36, 38, 59, 62, 64–73].

A barrier from the literature that was not upheld was the negative effects of CDSS on patient communication [36, 61, 64]. Our study participants were favourable that the tool could enhance communication through providing standard wording and removing pregnant patients’ perceptions that they were being asked because their GP ‘judged’ them as someone who consumed alcohol.

A major theme raised by our clinician participants was that while the integration of an alcohol screening prompt would be likely to increase screening for antenatal alcohol use in general practice settings (notwithstanding the additional barriers in providing preconception advice), there remain systemic barriers to consistent and universal screening that need to be addressed. Lack of time, even with a software prompt, was a major barrier and reform to how general practice antenatal care and specifically alcohol screening during pregnancy are funded through the MBS was considered key. Other Australian research with GPs has flagged that within a fee-for-service model of primary care, patients may be reluctant to bear the costs of a more prolonged consultation for advice that they had not specifically sought [57]. With additional funding for primary care promised by Australian state and federal government budgets [74, 75], lobbying for Medicare reform to antenatal consultation rebates is timely.

The design recommendations for ‘wished for’ features for a screening tool integrated into clinical practice software, developed through this consultation with GPs, received a very high level of agreement during the process of sense-testing. Features included: use of the tool triggered when a GP documents a current pregnancy in the EMR, accessible outside of automated prompting, generation of risk scores be informational and incorporated into the relevant data fields without overwriting prior scores (to establish longitudinal trends), enable streamlining of data collection and collation to avoid duplication of effort in clinical tasks (e.g. calculated scores be encoded in such a way that they can auto-populate relevant fields in health summaries, electronic referrals and shared maternity care records (with patient consent)), and the data be accessible to GPs for their own reporting and quality improvement activities.

End-user co-design was unanimously agreed as important, as was the delivery of in-education and training. Co-design of CDSS, including creation, design, and evaluation, is a widely accepted practice, as is ongoing evaluation to monitor clinician performance and the use of the tool over time, and the establishment of a knowledge management process to maintain the quality and integrity of the evidence-based content are understood to minimise error, manage risk, and promote user confidence in the system [35–38, 59–62, 64, 65, 69–71, 76–78].

A major recommendation arising was that rather than the tool screening for alcohol only, that it be a multifunctional antenatal screening tool incorporating screens for nicotine and other substance use, mental health, domestic and family violence and other information that is important to support a healthy pregnancy. When this recommendation was sense-tested, 18 of 19 responding clinicians agreed or strongly agreed with this (Table 4 and Supplement VII). If developed as a multifunctional tool, GPs wanted it to have functionality to complete only part of the screen, save progress and return to the screen at another time.

As an adjunct to the integration of a screening tool into general practice clinical software, the idea of using pre-consultation questionnaires was raised as a possible avenue for increasing routine screening of people who are pregnant or planning pregnancy in a way that enables GPs to make better use of their short consultation times with patients and address the results of the screening within the consultation. Such questionnaires could be deployed by organisations that partner with clinical software vendors (e.g., triggered at time of booking when patient states reason for visit) or having patients complete the questionnaire in the GP waiting room (via a smart phone/tablet or paper copy). Some GPs already used this to deploy common mental health screening tools. For alcohol screening, the Grog Survey App [46–48] and ASSIST [49] tools, generally too lengthy to use in GP consultations, provide more information to clinicians and so have potential utility in the pre-consultation questionnaire space. If using organisations that partner with the clinical software vendor to deploy the screening tool(s) used by the general practice, the preferred mechanism was to have the results of screening fed back into the general practice clinical software fields. This is not always possible due to privacy arrangements. Instead, the results may be provided as a PDF supplement that attaches to the patient’s EMR.

Many vendors have already integrated the standard AUDIT-C tool into their general GP software. However, none of them had made it (or any other alcohol screening tool) accessible from their obstetrics and gynaecology modules. Interviewed vendor representatives had varying insights into how long it would take to integrate a tool such as AUDIT-C for pregnancy into their clinical software, from days to over a year. Smaller vendors reported being able to respond to development requests faster than large vendors that described long clinical and governance review processes and already full schedules for updates planned out for the year ahead (updates for on-premises software). The feasibility of enacting changes within some of the ‘smaller’ (lesser market share) clinical software systems is therefore high with their lower cost and shorter timeframes for deployment (especially in cloud-based products); so, targeting a small vendor to help develop, pilot, deploy and evaluate a new screening tool could be worthwhile.

Whether a standalone alcohol screening tool or a multifunctional antenatal tool that incorporates alcohol screening is developed and implemented, the development costs and timeframe for integration between the two, from a vendor’s perspective, may not be far different. Difference lies in the time required to co-design and test (including for cultural appropriateness) a multifunctional/holistic antenatal screening tool.

The multiple clinical software packages used in Australian general practice and lack of enforced standards across software platforms [79] means that difference in the software’s underlying architecture will translate to technical differences to the back end of the antenatal screening tools and differences in the user interface. This is a fundamental barrier to the use and re-use of a ‘blueprint’ set of specifications for the development and integration of a clinical decision support tool for alcohol screening of pregnant women across all clinical management software products. Nonetheless, with two general practice software vendors having the majority of the market share, it is feasible to implement a tool that could be used by the majority of general practices in Australia. The clinical software systems used in general practice are not the same as the systems used in secondary and tertiary health care settings in Australia. A different piece of work would need to be undertaken to determine the needs and feasibility of integrating alcohol screening tools for use during pregnancy and preconception care into secondary and tertiary healthcare settings, although lessons could be learned from this work.

### Strengths and limitations

To our knowledge this study is the first in an Australian context to comprehensively canvas clinician and software vendor views on the integration of a screening tool into clinical practice software, specifically for review of alcohol use during pregnancy. Despite an earlier study concluding that such prompts could be a strong mechanism to increase rates of alcohol screening, [30] when presented with a prototype and asked to think through how they would use it, the barriers and enablers of alcohol screening in general practice became more nuanced. Our findings point to the urgent need for health resourcing reform, to enable GPs to have more time with pregnant patients to ensure comprehensive care, including early and regular alcohol screening. The breadth of our recommendations is a strength, but despite wide dissemination of the invitation to ‘sense test’ the recommendations, the stakeholder response rate for sense-testing was poor. This might reflect a lack of concern among primary care clinicians, or the very issue we have highlighted, clinician’s lack of time. Consequently, there are gaps in our sample, especially for the ‘sense-testing’. The low response rate and lack of representativeness means that further stakeholder consultation, especially for remote and/or culturally and linguistically diverse populations, should be undertaken before recommendations arising from this work (Table 2) are adopted.

Another limitation was that when we conducted the literature review, we restricted our search to screening instruments that were used during pregnancy and focussed on alcohol use; however, in our subsequent interviews with primary care clinicians, there was a clear preference for multifaceted screening tools that collected information about a broader array of psychosocial factors impacting on health and wellbeing. This suggests that there may be a stronger preference from clinicians for one of the multifaceted questionnaires that we had excluded from our prior review, such as the extended Antenatal Risk Questionnaire (ANQR), which encompasses risk factors for psychological distress in the perinatal period, as well as screening for domestic violence and alcohol/drug use, or the Indigenous Risk Impact Screen (IRIS), which is a screen for mental health risks and problem alcohol and other drug use validated in Aboriginal and Torres Strait Islander populations in Australia [50, 80].

A challenge we have noted, not identified by the interviewed stakeholders, was discussion always centred around pregnant women and did not account for pregnant people who do not identify as women. This is a relevant consideration both from a clinical care and a software perspective. Many of the software systems offer tools and modules in an individual patient’s EMR based on how their sex or gender is recorded in the software (for instance, patients documented as male will not receive prompts for overdue cervical screening tests or have access to obstetric modules). Where digital tools use algorithms like these to determine which prompts will be generated for which patients, there is the potential to reinforce disparities in care delivery for birth parents who do not identify as women. There must be a mechanism in the software for clinicians to access appropriate screening tools for their patients based on their clinical needs rather than their recorded sex or gender.

## Conclusions

The integration of a tool for screening for alcohol use among people who are pregnant or planning pregnancy into general practice clinical software is feasible; however, a multifunctional antenatal screening tool, incorporating other psychosocial elements, was considered more useful than a stand-alone alcohol screening tool. Codesign is needed with vendors and end-users to develop an acceptable tool that can be widely implemented so to facilitate routine alcohol screening among those who are pregnant or planning pregnancy, attending primary care consultations. The lack of appropriate funding for GPs to conduct preconception and prenatal alcohol screening is a significant barrier to improving rates of screening. A funding scheme that aligns with clinical practice guidelines could support and incentivise GPs to screen for alcohol use early and often during pregnancy.

## Supplementary Information

Supplementary Material 1.

## Data Availability

The datasets used and/or analysed during the current study are available from the corresponding author on reasonable request.

## Abbreviations

ASSIST: Alcohol, Smoking and Substance Involvement Screening Test
AUDIT-C: Alcohol Use Disorder Identification Test-Consumption subset (and AUDIT-C for pregnancy)
BP: Best Practice (clinical software package)
CDSS: Clinical Decision Support System
EMR: Electronic Medical Record
FARE: Foundation for Alcohol Research and Education
FASD: Foetal Alcohol Syndrome Disorder
GP: General practitioner
HPSR: Health Policy and Systems Research
IRIS: Indigenous Risk Impact Screen
MD: MedicalDirector (clinical software package)
RACGP: Royal Australian College of General Practitioners
