# Supplementary material for: The feasibility of integrating an alcohol screening clinical decision support tool into primary care clinical software: a review and Australian key stakeholder study
Source: BMC Prim Care. 2024 Dec 2;25:408. doi: 10.1186/s12875-024-02662-4 (PMC11610076; doi:10.1186/s12875-024-02662-4)
Supplement: Supplementary file 1 — Supplementary Material 1. [file 12875_2024_2662_MOESM1_ESM.pdf]

Supplementary files for "The feasibility of integrating an alcohol screening clinical decision support tool into primary care clinical software: a review and Australian key stakeholder study" by Canaway et al. 2024 *BMC Primary Care*

Supplement I: Clinical software systems used in Australian general practice 1
Supplement II: Alcohol use screening tools for health care settings 2
Supplement III: Literature review methodology for questions A, B, C.. 4
Supplement IV: Prototype alcohol screening tool.. 5
Supplement V: Interview question guide 10
Supplement VI: Recommendation feedback tool 11
Supplement VII: Screening tool design and implementation recommendations 12

Supplement I: Clinical software systems used in Australian general practice

	Software product	Vendor	Comments
1	BP Premier	Best Practice Software P/L	On premises. One of the most widely used products in Australia. https://bpsoftware.net/
2	Clinic to Cloud	Clinic to Cloud P/L	Cloud-based. https://www.clinictocloud.com/
3	Communicare	Telstra health	On premises. The most widely used product in Aboriginal Medical Services. https://www.communicare.org.au/
4	Direct Control	Connect Direct	Hybrid, desktop and browser apps. https://www.directcontrol.com.au/
5	Genie	Genie Solutions P/L	Vendor reported it is rarely used in general practice, mostly used by specialist medical clinics. https://www.geniesolutionssoftware.com.au/
6	Helix	Telstra Health	Cloud-based product. https://www.medicaldirector.com/products/helix
7	MD Clinical	Telstra Health (MedicalDirector)	On-premises product, one of the most widely used products in Australia. https://www.medicaldirector.com/
8	Medilink	Medilink	Hybrid. Desktop and cloud. https://www3.medilink.com.au/
9	MediRecords	MediRecords	Cloud-based. https://medirecords.com/
10	Medtech 32 / Evolution	Medtech Global Ltd	Hybrid, on-premises or cloud. Widely used in New Zealand, released in Australian in 2002. https://medtechglobal.com/au/
11	MMEx	ISA Healthcare Solutions	Cloud-based. Used in Aboriginal Medical Services in some parts of Australia and other general practices. https://www.isahealthcare.com/mmex
12	PCIS (Primary Care Information System)	Northern Territory Department of Health	Used in the Northern Territory of Australia in rural and remote health clinics.
13	The Practice	Godbar Software / Frayron P/L	On premise product. https://www.thepracticesoftware.com/
14	PrimaryClinic	Global Health Ltd	Hybrid: has cloud hosted option. https://www.primaryclinic.com.au/global-health/
15	Profile	Intrahealth Australia Ltd	On premises and cloud-based products. https://intrahealth.com/
16	Stat	Stat Health Systems	Includes cloud hosted option. https://stathealth.com.au/
17	Sunrise EMR & PAS	South Australia Health	https://www.sahealth.sa.gov.au/wps/wcm/connect/public+content/sa+health+internet/clinical+resources/clinical+information+systems/
18	Zedmed	Zedmed P/L	On premise or cloud version. https://www.zedmed.com.au/

Note: Locally installed 'on premises' software is on a computer at the general practice or on a server servicing the practice. Cloud-based products have the software hosted on the vendor's server with businesses accessing it via their web browser and therefore able to access it from any computer with internet access.

Supplement II: Alcohol use screening tools for health care settings

	Tool	Comments
1a	**AUDIT**	A 10-item screening questionnaire Alcohol Use Disorder Identification Test (AUDIT) developed by the World Health Organization. Includes questions about patterns of alcohol consumption, as well as about behavioural and attitudinal factors that suggest higher risk alcohol use and dependence. Has lower sensitivity for stop-start drinking patterns. [1]
1b	**AUDIT-C**	A 3-question subset of AUDIT about the frequency and quantity of alcohol use. Widely used and validated in the Australian context and incorporated into a range of general practice clinical management systems in Australia, including Aboriginal and Torres Strait Islander health settings. [2] When used in Aboriginal health settings, assistance often required to convert non-standard measures of alcohol intake into standard drinks. [3, 4]
1c	**AUDIT-C for pregnancy**	The same 3-questionnaire as AUDIT-C, but asks specifically about alcohol use in pregnancy, and has variable cut-offs for different risk thresholds to reflect that any level of alcohol consumption in pregnancy confers some risk. [5] May fail to identify women who limit their drinking during pregnancy to 'special occasions', suggesting that an additional supplementary question may be required. [6]
2	**T-ACE**	A 4-item alcohol screening tool, the first developed and validated specifically for use in obstetric settings (United States). Asks about alcohol tolerance rather than alcohol use with the rationale that many women may not realise that higher tolerance typically reflects higher consumption and as such would feel less stigma about their response. Widely used internationally but generalisability to an Australian setting is limited. [7] Not sensitive for detection of low levels or infrequent alcohol use. [8-12]
3	**4-Ps Plus**	5-questions to address under-disclosure of alcohol use in pregnancy, developed and validated for antenatal care settings. Asks about alcohol use in the month before pregnancy (drinking prior to pregnancy is a strong predictor of use in pregnancy). High sensitivity for detection of alcohol use in pregnancy, while reducing the burden of disclosure on patients. It is quick to use. A significant disadvantage of the 4-Ps Plus tool is that it is copyrighted and requires payment of a licence to use. [13]
4	**One Question Screen**	A single question tool that may have value in the Australian primary care context: 'When was your last drink?' to triage pregnant patients depending on whether they respond as a non-drinker, that their last drink was before pregnancy recognition, or their last drink was after pregnancy recognition. Developed and validated in health settings in the Congo. In the event of a positive screen it requires the clinician to ask further questions to quantify alcohol consumption patterns. [14]
5	**ASSIST**	8 item Alcohol, Smoking and Substance Involvement Screening Test (ASSIST) tool to identify and quantify alcohol and substance use, including in pregnancy. Includes consumption metrics and indicators of dependence and addiction. Estimated to take 5-10 minutes to complete. [15]
6	**SMAST**	A 13-item questionnaire, the Short Michigan Alcoholism Screening Test (SMAST) to detect alcohol use disorders, explores alcohol use across the lifetime. Abbreviated Michigan Alcoholism Screening Test (**MAST**). Used in some antenatal contexts, [12, 16, 17] but poor sensitivity for detecting low levels of alcohol use in pregnancy [8].
7	**CAGE**	4-questions to assess perception of drinking behaviours, optimised for detecting alcohol use **disorders** in non-pregnant populations. Does not quantify levels or patterns of consumption and in antenatal settings it is only sensitive to detecting higher levels of consumption and dependence. [12, 17] One of the earliest alcohol screening tools developed with the intent of removing stigma around disclosure of alcohol use [18]. An adapted version called CAGE-AID is used to screen for other substance use.
8	**TWEAK**	5-questions including from T-ACE, MAST and CAGE. [19, 20] Validated for pregnancy. One of the more widely used tools internationally but only likely to detect higher levels of alcohol consumption and dependence. [12, 16, 21] Does not quantify levels or patterns of alcohol consumption, so if positive, it requires clinicians to take further history.
9	**NET**	3-questions (Normal drinker, Eye-opener, Tolerance) for use in antenatal contexts [22]. Has high sensitivity and specificity for high-risk drinking but is unlikely to detect low and moderate alcohol use. [23]. Has been critiqued as of high risk of bias due to asking whether the person considers themselves to be a 'normal drinker' [12].
10	**Grog Survey App**	A 'patient-facing' rather than clinician directed screening tool. Validated for use specifically in Aboriginal and Torres Strait Islander health settings. Not validated for use in pregnancy. A culturally sensitive resource that may inform the development of clinician resources. Based on a modified Finnish model, which takes a narrative approach to screening. More time consuming than the AUDIT-C, which limits its appropriateness in many general practice contexts. [4, 24, 25]
11	**IRIS***	Indigenous Risk Impact Screen (IRIS) for determining the presence of alcohol, drug and mental health risk in Indigenous adult Australians, and the scores that discriminate between the presence and absence of risk. Includes AUDIT, the Severity of Dependence Scale and the Leeds Dependence Questionnaire, and two mental health screens. [26, 27]

* Note: IRIS was not picked up in our initial review because alcohol screening is but one of several measures and it was not flagged as a tool used for pregnancy or prenatal screening. We have added it for completeness.

Alcohol use screening tools for health care settings: References

1. Babor TF, Higgins-Biddle JC, Saunders JB, Monteiro MG. AUDIT: the Alcohol Use Disorders Identification Test: Guidelines for Use in Primary Care (second edition) 2001 21 December 2023 [cited 2023 21 December]:[39 p.]. Available from: https://www.who.int/publications/i/item/WHO-MSD-MSB-01.6a.
2. Conigrave JH, Lee KSK, Haber PS, Vnuk J, Doyle MF, Conigrave KM. More than three times as many Indigenous Australian clients at risk from drinking could be supported if clinicians used AUDIT-C instead of unstructured assessments. Addiction Science & Clinical Practice. 2022;17(1):23. doi:10.1186/s13722-022-00306-5
3. Lee KSK, Conigrave JH, Callinan S, Wilson S, Room R, Perry J, et al. Asking about the last four drinking occasions on a tablet computer as a way to record alcohol consumption in Aboriginal and Torres Strait Islander Australians: a validation. Addiction science & clinical practice. 2019;14(1):15-. doi:10.1186/s13722-019-0148-2
4. Lee KSK, Conigrave JH, Wilson S, Perry J, Callinan S, Room R, et al. Short screening tools for risky drinking in Aboriginal and Torres Strait Islander Australians: modified AUDIT-C and a new approach. Addiction science & clinical practice. 2019;14(1):22-. doi:10.1186/s13722-019-0152-6
5. FARE. Using the AUDIT-C for assessment of alcohol use in pregnancy: a resource for health professionals. Every Moment Matters, Foundation of Alcohol Research and Education (FARE); n.d. [cited 2023 28 May]. Available from: https://everymomentmatters.org.au/resources/?type=fact-sheet.
6. Tsang TW, Kingsland M, Doherty E, Anderson AE, Tully B, Crooks K, et al. Predictors of alcohol use during pregnancy in Australian women. Drug and Alcohol Review. 2022;41(1):171-81. doi:10.1111/dar.13330
7. Chiodo LM, Sokol RJ, Delaney-Black V, Janisse J, Hannigan JH. Validity of the T-ACE in pregnancy in predicting child outcome and risk drinking. Alcohol. 2010;44(7-8):595-603. doi:10.1016/j.alcohol.2009.08.009
8. Chang G, Wilkins-Haug L, Berman S, Goetz MA, Behr H, Hiley A. Alcohol Use and Pregnancy: Improving Identification. 1998.
9. Sokol RJ, Martier SS, Ager JW. The T-ACE questions: practical prenatal detection of risk-drinking. Am J Obstet Gynecol. 1989;160(4):863-8; discussion 8-70. doi:10.1016/0002-9378(89)90302-5
10. Chang G. Alcohol-screening instruments for pregnant women. Alcohol Res Health. 2001;25(3):204-9.
11. Chang G. Screening and brief intervention in prenatal care settings. Alcohol Res Health. 2004;28(2):80-4.
12. Dozet D, Burd L, Popova S. Screening for Alcohol Use in Pregnancy: a Review of Current Practices and Perspectives. International Journal of Mental Health and Addiction. 2021. doi:10.1007/s11469-021-00655-3
13. Chasnoff IJ, McGourty RF, Bailey GW, Hutchins E, Lightfoot SO, Pawson LL, et al. The 4P's Plus© screen for substance use in pregnancy: Clinical application and outcomes. Journal of Perinatology. 2005;25(6):368-74. doi:10.1038/sj.jp.7211266
14. Williams AD, Nkombo Y, Nkodia G, Leonardson G, Burd L. Prenatal alcohol exposure in the Republic of the Congo: Prevalence and screening strategies. Birth Defects Research Part A - Clinical and Molecular Teratology. 2013;97(7):489-96. doi:10.1002/bdra.23127
15. Humeniuk R, Henry-Edwards S, Ali R, Poznyak V, V.Montiero MG, World Health Organisation. The Alcohol, Smoking and Substance Involvement Screening Test (ASSIST): manual for use in primary care. 2010.
16. Russell M, Martier SS, Sokol RJ, Mudar P, Jacobson S, Jacobson J. Detecting risk drinking during pregnancy: a comparison of four screening questionnaires. Am J Public Health. 1996;86(10):1435-9. doi:10.2105/ajph.86.10.1435
17. Montag AC. Fetal alcohol-spectrum disorders: Identifying at-risk mothers. International Journal of Women's Health. 2016;8:311-23. doi:10.2147/IJWH.S85403
18. Ewing JA. Detecting alcoholism. The CAGE questionnaire. JAMA. 1984;252(14):1905-7. doi:10.1001/jama.252.14.1905
19. Russell M. New Assessment Tools for Risk Drinking During Pregnancy: T-ACE, TWEAK, and Others. Alcohol Health Res World. 1994;18(1):55-61.
20. Russell M, Bigler L. Screening for alcohol-related problems in an outpatient obstetric-gynecologic clinic. Am J Obstet Gynecol. 1979;134(1):4-12. doi:10.1016/0002-9378(79)90787-7
21. Praestegaard C, Kesmodel PS, Kesmodel US. Is TWEAK a valid screening questionnaire to identify alcohol risk drinkers among pregnant women in Denmark? Acta Obstet Gynecol Scand. 2018;97(4):483-90. doi:10.1111/aogs.13314
22. Bottoms S, Martier S, Sokol R. Refinements in screening for risk drinking in reproductive-aged women: The "NET" results. Alcohol Clin Exp Res. 1989;13(2a):339.
23. Burns E, Gray R, Smith LA. Brief screening questionnaires to identify problem drinking during pregnancy: a systematic review. Addiction. 2010;105(4):601-14. doi:10.1111/j.1360-0443.2009.02842.x
24. Lee KK, Conigrave JH, Al Ansari M, Wilson S, Perry J, Zheng C, et al. Acceptability and feasibility of a computer-based application to help Aboriginal and Torres Strait Islander Australians describe their alcohol consumption. Journal of Ethnicity in Substance Abuse. 2021;20(1):16-33. doi:10.1080/15332640.2019.1579144
25. Zheng C, Conigrave JH, Conigrave KM, Wilson S, Perry J, Chikritzhs TN, et al. Patterns of drinking in Aboriginal and Torres Strait Islander peoples as self-reported on the Grog Survey App: A representative urban and remote sample. Drug and Alcohol Review. 2022;41(1):114-24. doi:10.1111/dar.13333
26. Schlesinger CM, Ober C, McCarthy MM, Watson JD, Seinen A. The development and validation of the Indigenous Risk Impact Screen (IRIS): a 13-item screening instrument for alcohol and drug and mental health risk. Drug and Alcohol Review. 2007;26(2):109-17. doi:https://doi.org/10.1080/09595230601146611
27. Islam MM, Oni HT, Lee KSK, Hayman N, Wilson S, Harrison K, et al. Standardised alcohol screening in primary health care services targeting Aboriginal and Torres Strait Islander peoples in Australia. Addiction Science and Clinical Practice: BioMed Central Ltd.; 2018.

Supplement III: Literature review methodology for questions A, B, C.

Search strategy for questions A and B:

A. Which patients should be screened for alcohol use when pregnant or when planning a pregnancy?
B. What screening tools for alcohol use during pregnancy have been validated for use in primary care settings, both nationally and internationally?

Manual searches of MEDLINE (Web of Science: 1900-2022) and Scopus (1996-2022) databases were conducted by LD in December 2022. The search was limited to English-language studies published up to December 2022. The inclusion of 'primary care' search terms were too restrictive, as many of the studies and papers discuss use of the tools in antenatal care settings without specifying whether these are primary or secondary care settings, and many of the screening tools identified do not have validation studies specifically for primary care settings. We therefore included the broader term of healthcare to allow for inclusion of literature about alcohol screening in any care setting where antenatal care was being delivered.
1431 articles were captured in the search. We also captured papers with an expanded search by using snowballing from reference lists of key articles, and purposive searches for key guidelines. Of all evidence captured and after review of data, 58 relevant articles, guidelines and reports were used to inform review question A and B.

Example search terms:
alcohol OR ethanol OR drink* (All Fields) AND pregnan* OR antenatal OR prenatal (All Fields) AND healthcare OR primary AND care OR general AND practi* OR gp OR doctor OR physician OR family AND physician (All Fields) AND identif* OR detect* OR screen* (All Fields)

Search strategy for question C:

C. What clinical software management software systems are in use in Australian general practice?

Several methods were used to develop a list of EMR software systems and vendors. Firstly, we reviewed the existing literature on EMR systems to identify commonly mentioned vendors located in Australia. Secondly, we cross-referenced these findings with a vendor list from My Health Record (B2B) Register of Conformance list accessed via the Australian Digital Health Agency (ADHA) website to ensure comprehensiveness.* Last, we conducted searches online using Google search engine to identify any additional vendors and assessed their relevance to GP medical EMR.

Note: The register of conformity lists software products and the versions that have been assessed for conformance with national digital health requirements. This includes the ability to view a My Health Record, upload a shared health summary, upload prescriptions, provide assisted registration, and more. Software developers must declare the conformance of their products to be included in the Register.

* Australian Digital Health Agency. My Health Record (B2B) Register of Conformance Canberra: Australian Government, Australian Digital Health Agency; 2023 [cited 2023 13 January]. Available from: https://www.digitalhealth.gov.au/about-us/policies-privacy-and-reporting/registers.

Supplement IV: Prototype alcohol screening tool

The prototype clinical decisions support / screening tool as shown on the following pages was developed by Dr Libby Dai to be a discussion prompt for interviewees. The prototype was provided to each interview participant and discussed during each interview.

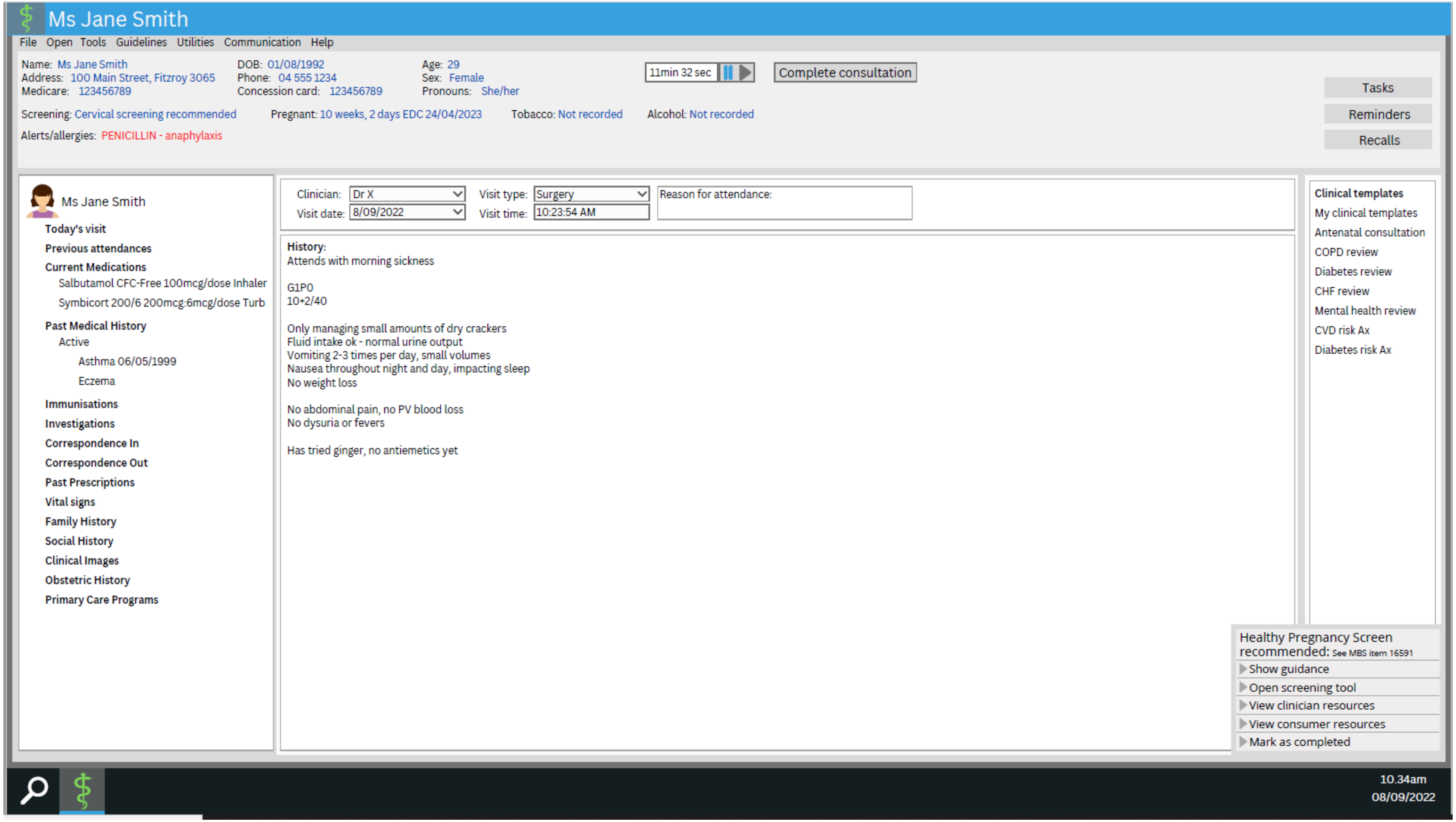

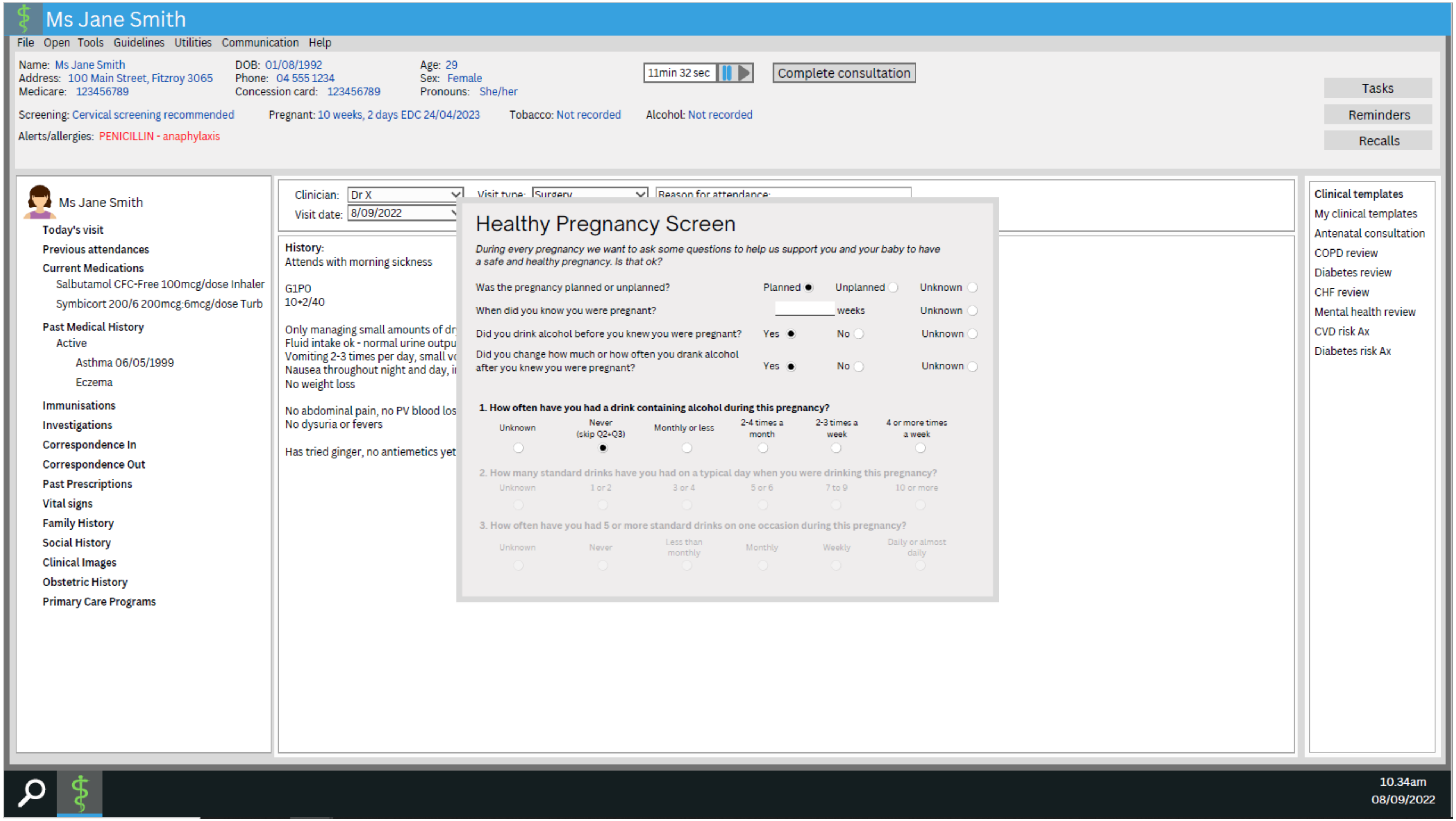
Ms Jane Smith
File Open Tools Guidelines Utilities Communication Help
Name: Ms Jane Smith
Address: 100 Main Street, Fitzroy 3065
Medicare: 123456789
DOB: 01/08/1992
Phone: 04 555 1234
Concession card: 123456789
Age: 29
Sex: Female
Pronouns: She/her
11min 32 sec
Complete consultation
Tasks
Reminders
Recalls
Screening: Cervical screening recommended
Pregnant: 10 weeks, 2 days EDC 24/04/2023
Tobacco: Not recorded
Alcohol: Not recorded
Alerts/allergies: PENICILLIN - anaphylaxis
Ms Jane Smith
Today's visit
Previous attendances
Current Medications
Salbutamol CFC-Free 100mcg/dose Inhaler
Symbicort 200/6 200mcg:6mcg/dose Turb
Past Medical History
Active
Asthma 06/05/1999
Eczema
Immunisations
Investigations
Correspondence In
Correspondence Out
Past Prescriptions
Vital signs
Family History
Social History
Clinical Images
Obstetric History
Primary Care Programs
Clinician: Dr X
Visit date: 8/09/2022
Visit type: Surgery
Reason for attendance:
History:
Attends with morning sickness
G1P0
10+2/40
Only managing small amounts of dr
Fluid intake ok - normal urine outpu
Vomiting 2-3 times per day, small vo
Nausea throughout night and day, i
No weight loss
No abdominal pain, no PV blood los
No dysuria or fevers
Has tried ginger, no antiemetics yet
Healthy Pregnancy Screen
During every pregnancy we want to ask some questions to help us support you and your baby to have a safe and healthy pregnancy. Is that ok?
Was the pregnancy planned or unplanned? Planned Unplanned Unknown
When did you know you were pregnant? weeks Unknown
Did you drink alcohol before you knew you were pregnant? Yes No Unknown
Did you change how much or how often you drank alcohol after you knew you were pregnant? Yes No Unknown
1. How often have you had a drink containing alcohol during this pregnancy?
Unknown
Never (skip Q2+Q3)
Monthly or less
2-4 times a month
2-3 times a week
4 or more times a week
2. How many standard drinks have you had on a typical day when you were drinking this pregnancy?
Unknown
1 or 2
3 or 4
5 or 6
7 to 9
10 or more
3. How often have you had 5 or more standard drinks on one occasion during this pregnancy?
Unknown
Never
Less than monthly
Monthly
Weekly
Daily or almost daily
Clinical templates
My clinical templates
Antenatal consultation
COPD review
Diabetes review
CHF review
Mental health review
CVD risk Ax
Diabetes risk Ax
10.34am
08/09/2022

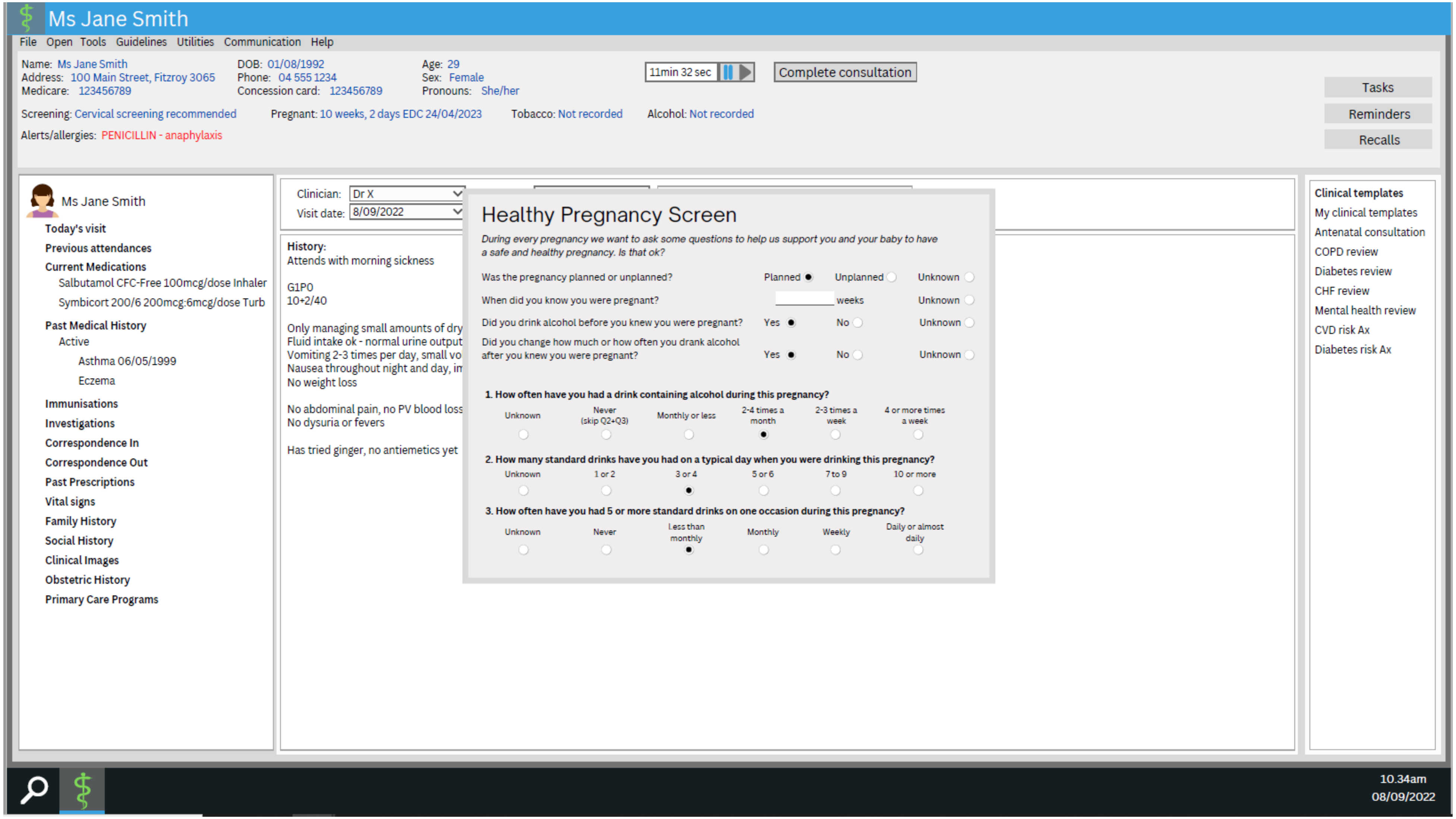
Ms Jane Smith
File Open Tools Guidelines Utilities Communication Help
Name: Ms Jane Smith
Address: 100 Main Street, Fitzroy 3065
Medicare: 123456789
DOB: 01/08/1992
Phone: 04 555 1234
Concession card: 123456789
Age: 29
Sex: Female
Pronouns: She/her
11min 32 sec
Complete consultation
Tasks
Reminders
Recalls
Screening: Cervical screening recommended
Pregnant: 10 weeks, 2 days EDC 24/04/2023
Tobacco: Not recorded
Alcohol: Not recorded
Alerts/allergies: PENICILLIN - anaphylaxis
Ms Jane Smith
Today's visit
Previous attendances
Current Medications
Salbutamol CFC-Free 100mcg/dose Inhaler
Symbicort 200/6 200mcg:6mcg/dose Turb
Past Medical History
Active
Asthma 06/05/1999
Eczema
Immunisations
Investigations
Correspondence In
Correspondence Out
Past Prescriptions
Vital signs
Family History
Social History
Clinical Images
Obstetric History
Primary Care Programs
Clinician: Dr X
Visit date: 8/09/2022
History:
Attends with morning sickness
G1P0
10+2/40
Only managing small amounts of dry
Fluid intake ok - normal urine output
Vomiting 2-3 times per day, small vo
Nausea throughout night and day, in
No weight loss
No abdominal pain, no PV blood loss
No dysuria or fevers
Has tried ginger, no antiemetics yet
Healthy Pregnancy Screen
During every pregnancy we want to ask some questions to help us support you and your baby to have a safe and healthy pregnancy. Is that ok?
Was the pregnancy planned or unplanned? Planned Unplanned Unknown
When did you know you were pregnant? weeks Unknown
Did you drink alcohol before you knew you were pregnant? Yes No Unknown
Did you change how much or how often you drank alcohol after you knew you were pregnant? Yes No Unknown
1. How often have you had a drink containing alcohol during this pregnancy?
Unknown Never (skip Q2+Q3) Monthly or less 2-4 times a month 2-3 times a week 4 or more times a week
2. How many standard drinks have you had on a typical day when you were drinking this pregnancy?
Unknown 1 or 2 3 or 4 5 or 6 7 to 9 10 or more
3. How often have you had 5 or more standard drinks on one occasion during this pregnancy?
Unknown Never Less than monthly Monthly Weekly Daily or almost daily
Clinical templates
My clinical templates
Antenatal consultation
COPD review
Diabetes review
CHF review
Mental health review
CVD risk Ax
Diabetes risk Ax
10.34am
08/09/2022

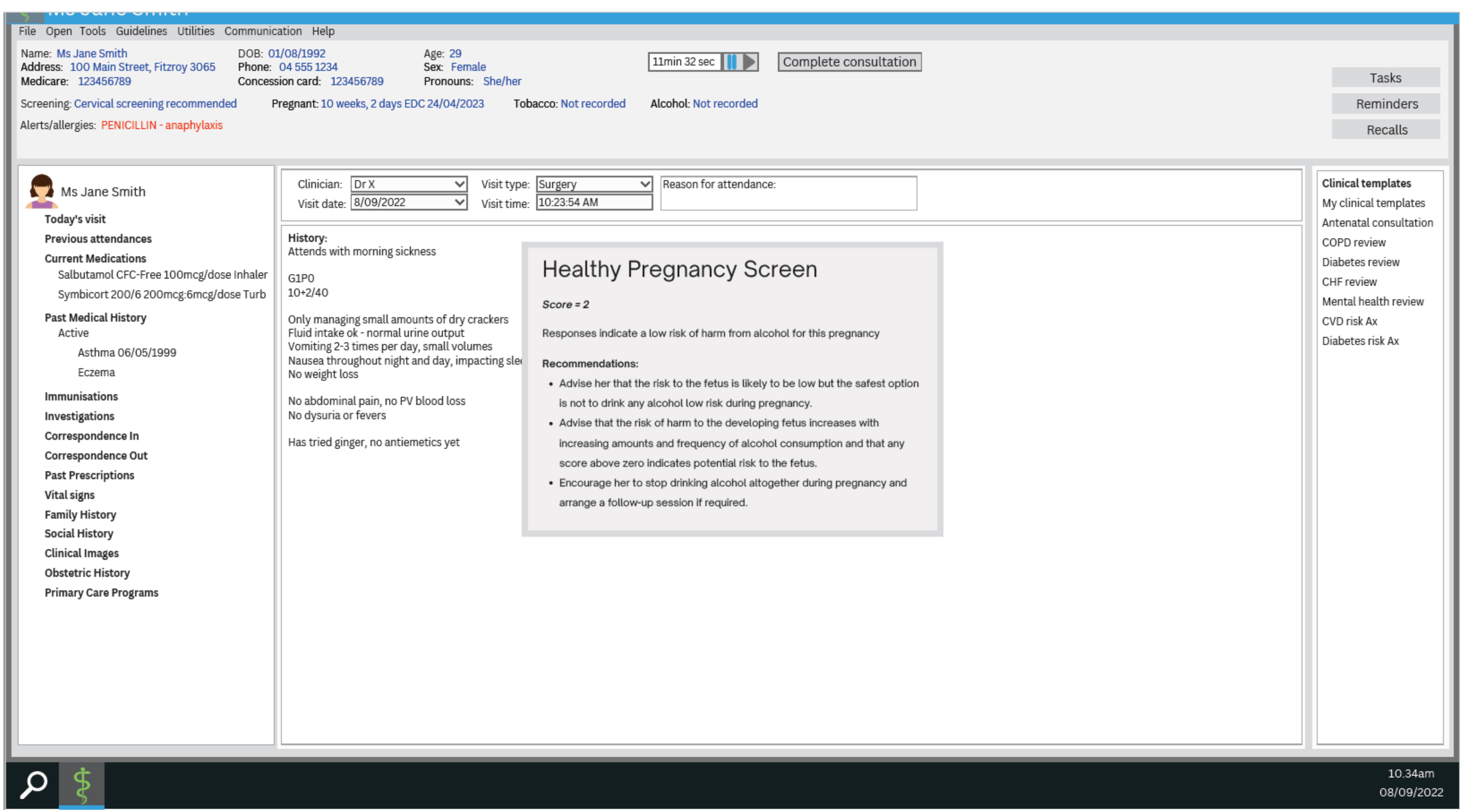
File Open Tools Guidelines Utilities Communication Help
Name: Ms Jane Smith
Address: 100 Main Street, Fitzroy 3065
Medicare: 123456789
DOB: 01/08/1992
Phone: 04 555 1234
Concession card: 123456789
Age: 29
Sex: Female
Pronouns: She/her
11min 32 sec
Complete consultation
Tasks
Reminders
Recalls
Screening: Cervical screening recommended
Pregnant: 10 weeks, 2 days EDC 24/04/2023
Tobacco: Not recorded
Alcohol: Not recorded
Alerts/allergies: PENICILLIN - anaphylaxis
Ms Jane Smith
Today's visit
Previous attendances
Current Medications
Salbutamol CFC-Free 100mcg/dose Inhaler
Symbicort 200/6 200mcg:6mcg/dose Turb
Past Medical History
Active
Asthma 06/05/1999
Eczema
Immunisations
Investigations
Correspondence In
Correspondence Out
Past Prescriptions
Vital signs
Family History
Social History
Clinical Images
Obstetric History
Primary Care Programs
Clinician: Dr X
Visit date: 8/09/2022
Visit type: Surgery
Visit time: 10:23:54 AM
Reason for attendance:
History:
Attends with morning sickness
G1P0
10+2/40
Only managing small amounts of dry crackers
Fluid intake ok - normal urine output
Vomiting 2-3 times per day, small volumes
Nausea throughout night and day, impacting sle
No weight loss
No abdominal pain, no PV blood loss
No dysuria or fevers
Has tried ginger, no antiemetics yet
Healthy Pregnancy Screen
Score = 2
Responses indicate a low risk of harm from alcohol for this pregnancy
Recommendations:
Advise her that the risk to the fetus is likely to be low but the safest option is not to drink any alcohol low risk during pregnancy.
Advise that the risk of harm to the developing fetus increases with increasing amounts and frequency of alcohol consumption and that any score above zero indicates potential risk to the fetus.
Encourage her to stop drinking alcohol altogether during pregnancy and arrange a follow-up session if required.
Clinical templates
My clinical templates
Antenatal consultation
COPD review
Diabetes review
CHF review
Mental health review
CVD risk Ax
Diabetes risk Ax
10.34am
08/09/2022

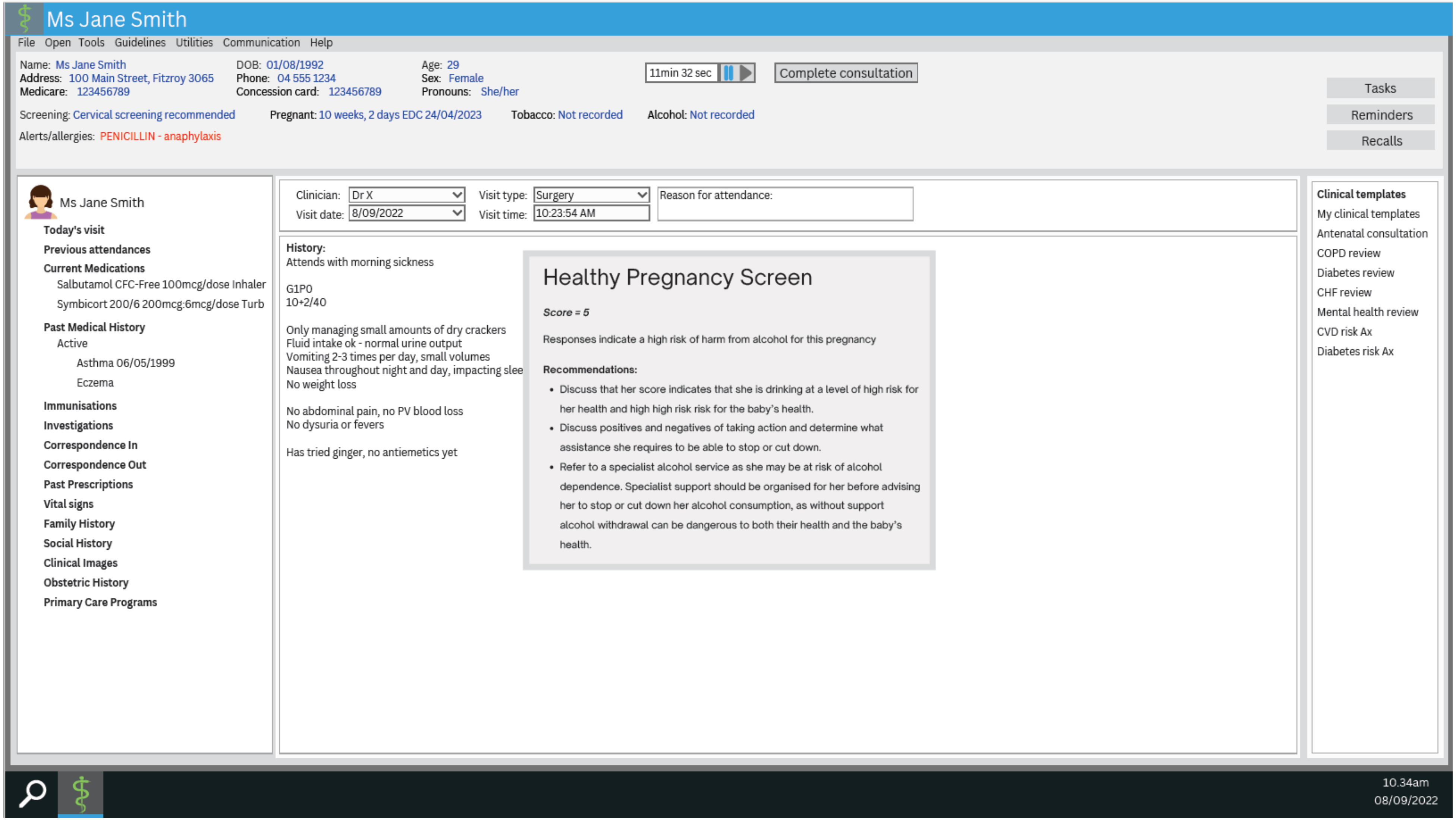
Ms Jane Smith
File Open Tools Guidelines Utilities Communication Help
Name: Ms Jane Smith
Address: 100 Main Street, Fitzroy 3065
Medicare: 123456789
DOB: 01/08/1992
Phone: 04 555 1234
Concession card: 123456789
Age: 29
Sex: Female
Pronouns: She/her
11min 32 sec
Complete consultation
Tasks
Reminders
Recalls
Screening: Cervical screening recommended
Pregnant: 10 weeks, 2 days EDC 24/04/2023
Tobacco: Not recorded
Alcohol: Not recorded
Alerts/allergies: PENICILLIN - anaphylaxis
Ms Jane Smith
Today's visit
Previous attendances
Current Medications
Salbutamol CFC-Free 100mcg/dose Inhaler
Symbicort 200/6 200mcg:6mcg/dose Turb
Past Medical History
Active
Asthma 06/05/1999
Eczema
Immunisations
Investigations
Correspondence In
Correspondence Out
Past Prescriptions
Vital signs
Family History
Social History
Clinical Images
Obstetric History
Primary Care Programs
Clinician: Dr X
Visit date: 8/09/2022
Visit type: Surgery
Visit time: 10:23:54 AM
Reason for attendance:
History:
Attends with morning sickness
G1P0
10+2/40
Only managing small amounts of dry crackers
Fluid intake ok - normal urine output
Vomiting 2-3 times per day, small volumes
Nausea throughout night and day, impacting slee
No weight loss
No abdominal pain, no PV blood loss
No dysuria or fevers
Has tried ginger, no antiemetics yet
Healthy Pregnancy Screen
Score = 5
Responses indicate a high risk of harm from alcohol for this pregnancy
Recommendations:
Discuss that her score indicates that she is drinking at a level of high risk for her health and high high risk risk for the baby's health.
Discuss positives and negatives of taking action and determine what assistance she requires to be able to stop or cut down.
Refer to a specialist alcohol service as she may be at risk of alcohol dependence. Specialist support should be organised for her before advising her to stop or cut down her alcohol consumption, as without support alcohol withdrawal can be dangerous to both their health and the baby's health.
Clinical templates
My clinical templates
Antenatal consultation
COPD review
Diabetes review
CHF review
Mental health review
CVD risk Ax
Diabetes risk Ax
10.34am
08/09/2022

Supplement V: Interview question guide

An overview of relevant interview questions was sent to each participant with the Plain Language Statement, Consent Form and Alcohol Screening Tool prototype.

A: Questions for everyone:

1. Briefly: your professional role and the type of organisation you work for.
2. Whether you are responding with the viewpoint of the organisation you represent or giving your own personal opinion.
9. Overall whether you think the development and deployment tools in clinical software to prompt for alcohol screening among pregnant (or planning) women is a good idea.
10. Other ways or ideas around ensuring that more women who are pregnant or planning pregnancy are routinely screened for alcohol use.
11. Any other considerations.

B: Questions for primary care clinicians / representative organisations / researchers:

3. What clinical software package you use in practice (if in practice).
4. Your current use of alcohol screening tools. When? Why? How do you use them?
5. Thoughts on available alcohol screening tools – their acceptability, benefits, barriers and pitfalls of their use.
6. Criteria you think are appropriate to initiate alcohol screening of women who are pregnant or planning pregnancy (include consideration of marginal communities: CALD, First Nations, LGBTQIA+, etc).
7. Perceived benefits and barriers, risks or pitfalls of integrating prompts / decision support tools for this purpose into general practice clinical management software.
8. Which clinical management software systems should be the priority to target if prompts for alcohol screening during pregnancy were integrated.

C: Questions for representatives of clinical software management organisations

3. Estimated market share of your EMR system in general practice and community care settings.
4. Do you have an alcohol screening tool integrated into your EMR? If YES, what prompts clinicians to use it? Do you have any idea how much it is used by clinicians?
5. Barriers and enablers to development and implementation of integrated prompts or decision support tools to encourage more routine use of alcohol screening tools.
6. The process that would need to be undertaken to integrate the tools.
7. Estimate of how long you think it would take to develop and implement prompts for alcohol screening into the GP clinical management software.
    a. The caveats to this time estimate
8. Estimate of costs associated with the development and implementation of the prompts / tool.

Supplement VI: Recommendation feedback tool

Hosted on the University of Melbourne's instance of Qualtrics, the feedback questionnaire commenced with a consent statement, outlined the objectives of the study, gave information about ethics approval and provided contact details for the researcher and ethics committee.

An eligibility check asked for respondents to choose one of the following as best describing themselves:

1. GP
2. Practice nurse
3. Other primary care clinician
4. Practice manager or other non clinical primary care staff
5. A representative of a primary care related Professional Association or College
6. A representative of an organisation associated with clinical software used in general practice
7. A research or health informatician
8. None of the above (this option exits you from the survey)

Options 1-4	Led to a further question: Do you have a role in an Aboriginal Medical Service?
Option 6	Led to a further question: Do you provide services/ software to Aboriginal Medical Services?
Option 7	Led to a further question: Do you have expertise in Aboriginal & Torres Strait Islander health research or data?

The following instruction was then given:

Instruction: In the context of Australian general practice and community care, please select how much you agree or disagree with each overall Recommendation. You may leave additional feedback in the box below each Recommendation.

When you have finished, please click on or tap the arrow at the bottom of the page to submit.

Each of the draft recommendations were then given, followed by a five point Likert scale and a text box for written feedback – as shown:

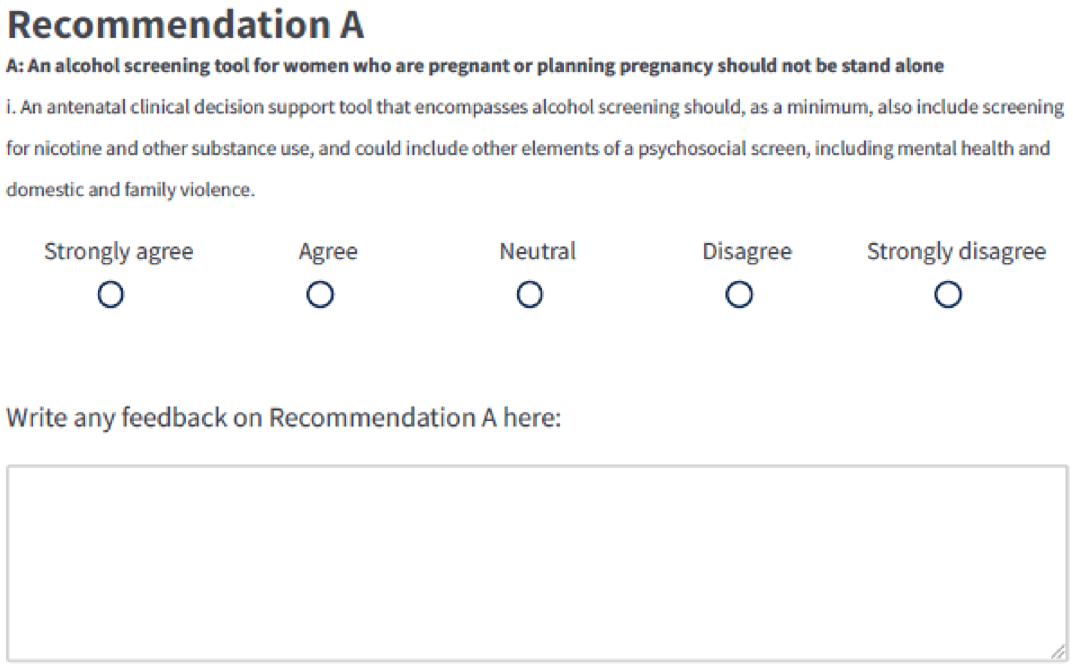

Recommendation A

A: An alcohol screening tool for women who are pregnant or planning pregnancy should not be stand alone

i. An antenatal clinical decision support tool that encompasses alcohol screening should, as a minimum, also include screening for nicotine and other substance use, and could include other elements of a psychosocial screen, including mental health and domestic and family violence.

Strongly agree | Agree | Neutral | Disagree | Strongly disagree

Write any feedback on Recommendation A here:

An additional text box was given for capturing 'Other general comments'.

Lastly, respondents were asked to choose their state, territory or jurisdiction from a drop-down list, which included 'National', and then to Submit their feedback.

Supplement VII: Screening tool design and implementation recommendations

A total of 54 free-text comments were left by 12 of the 22 'sense-testing' respondents. No respondent left a comment for every recommendation.

Screening tool design recommendations

A An alcohol screening tool for women who are pregnant or planning pregnancy should be multifunctional and holistic

i. An antenatal clinical decision support tool that encompasses alcohol screening should, as a minimum, also include screening for nicotine and other substance use, and could include other elements of a psychosocial screen, including mental health and domestic and family violence.

ii. As a multi-functional screening tool (including broad psychosocial screening elements) it should have the functionality for users to complete only part of the screen, save progress and return to the screen at another time. (Not relevant for an alcohol only screening tool.)

Sense test results, N=22

Strongly agree	Agree	Neutral	Disagree	Strongly disagree
**13**	5	3	1	Nil
Includes 1 x professional association / College representative		3 x vendors	1 x GP	

Comments from sense testing respondents: *"GPs are holistic care providers and not dealing with single issues." (Strongly agreeing GP)*

"Needs to be short enough so alcohol/smoking/vaping/drugs and maybe a couple of screening questions which could then direct more detailed psychosocial/DV assessment." (Strongly agreeing GP)

"These other issues are important, and for GPs who may be reluctant to explore alcohol due to stigma/cultural concerns, framing alcohol screening within a broader assessment framework will appeal." (Strongly agreeing GP)

"I think alcohol assessment and advice is part of a holistic assessment of every pregnant person. We don't do this in isolation in clinical practice." (Strongly agreeing GP)

"While your funders are alcohol-focused, GPs need to look at the big picture." (Strongly agreeing GP)

"May depend on the patient and might be more useful as a stand-alone tool depending on the scenario." (Disagreeing GP)

B Indicating 'Currently pregnant' in the clinical software triggers automated prompt for alcohol screening (+/- psychosocial screen)

i. Documenting a current pregnancy in the electronic medical record is an appropriate trigger point for the software to generate a prompt to consider conducting antenatal screening (e.g. as described at Recommendation A).

Sense test results, N=22

Strongly agree	Agree	Neutral	Disagree	Strongly disagree
**10**	9	2	1	Nil
Includes 1 x professional association / College representative	1 x vendor	2 x vendors	1 x GP	

Comments from sense testing respondents: *"Busy GPs will appreciate prompts." (Strongly agreeing GP)*

"We already get medication warning if pregnant - often for medications with much much less risk than alcohol. It makes sense this would be an important prompt." (Strongly agreeing GP)

"This should be repeated as pregnancy can be a time of turmoil." (Strongly agreeing GP)

"This needs to be tempered as it may not be appropriate to do it when it pops up. It must be able to be ignored or delayed. Otherwise GPs will resent doing it." (Agreeing GP) **Note:** This fits with Recommendation Dii.

"I think this may even be too late. We do most of this assessment at the first appointment in which case pregnancy will only be recorded at that appt. So the tool would need to be able to actively read the record during a consultation and recognise that this is a pregnancy appt in real-time. I'm not sure how we could flag it earlier, perhaps if a preconception counselling or pregnancy planning is recorded in the record." (Disagreeing GP)

Note: The GP who disagreed may not have understood that indicating a woman as pregnant would automatically trigger the decision support tools for alcohol and other antenatal screening. This respondent was strongly approving of Recommendation D.

C Pre-consultation questionnaires may be used in addition to in-consultation screening

i. If the in-consultation screening tool includes broader psychosocial screening (e.g., as described at Recommendation A), mechanisms may be developed to offer such screening via pre-consultation questionnaires (enabling GPs to make best use of short consultation times).

Sense test results, N=10 *(recommendation added to the feedback survey - seen only by the final 10 respondents):*

Strongly agree	Agree	Neutral	Disagree	Strongly disagree
**4**	4	2	Nil	Nil
		1 x vendor, 1 x other primary care clinician		

Comments from sense testing respondents: *"I would strongly recommend a software incorporated tool as above. I have a self created tool/questionnaire and hard copy I then give to my patients, which goes through similar issues and I go through it with my pregnant patients at the first appt and then re visit any highlighted concerns throughout the pregnancy." (Agreeing GP)*
"Such a tool should be available via an online booking system that prompts to complete this if not already done when booking an antenatal appointment. E.g. Automed systems or Healthshare" (Strongly agreeing GP)
"This would be very helpful - timewise and for data management." (Agreeing GP)
"Yes makes sense and streamline the data collection." (Strongly agreeing GP)
"GPs should be considering all the determinants of poor health."(Strongly agreeing GP)

D The decision support tool should be easily accessible outside of automated prompting

i. The antenatal screening tool should be accessible to be used for opportunistic discussions with non-pregnant patients who are planning pregnancy or are seeking pre-conception advice without the software generating a prompt.

ii. The prompt to consider antenatal screening should be visible on the clinical desktop and be non-interruptive to clinical workflow; i.e. it should not require the clinician to interact with it unless they choose to.

Sense test results, N=22

Strongly agree	Agree	Neutral	Disagree	Strongly disagree
9	**13**	Nil	Nil	Nil
	Includes 3 x vendors, 1 x professional association / College representative			

Comments from sense testing respondents: *"The is a big opportunity to reduce alcohol-related harm during antenatal visits. In addition, there will be times in a consult that automatic prompts need to be closed and then accessed later in the consultation when this assessment is more appropriate. Access at this time would be needed." (Strongly agreeing GP)*
"We need to focus pre-conceptually and also consider the male partners (in heterosexual relationships)" (Strongly agreeing GP)
"Sometimes automated prompting may interrupt the flow of a consult and not be appropriate to use at that particular moment. Having the ability to dismiss it at the time but access it again later, at a more appropriate time, would be very helpful." (Strongly agreeing GP)
"Agree, this fits with my above comments that an active prompt once pregnancy is recorded might be too late and I do a lot of preconception/fertility appts so starting the assessment and education earlier is better." (Strongly agreeing GP)

E Generated risk scores should be informational and incorporated into relevant data fields without overwriting prior scores

i. The antenatal screening tool should generate scores to risk-stratify patients and provide advice on clinical management according to the current guidelines.

ii. Documentation of calculated antenatal screening tool scores should be automatic and encoded in such a way that previous results can be displayed in series to allow clinicians to compare scores at previous timepoints.

Sense test results, N=22:

Strongly agree	Agree	Neutral	Disagree	Strongly disagree
5	**13**	3	1	Nil
	Includes 2 x vendor, 1 x professional association / College representative	2 x GP, 1 x vendor	1 x GP	

Comments from sense testing respondents: *"We do need to see a timeline." (Strongly agreeing GP)*
"Useful to have a trend." (Agreeing GP)
"There's too much 'grey' in medicine to be black and white about advice. Advice should include a spectrum of options including guideline recommendations (don't drink) through to harm minimisation recommendations (if you must drink, here's how to reduce harm), plus avenues for referral/support for those screened at high risk/dependence (stopping may be dangerous)" (Agreeing GP)

"I think GPs are not so interested in risk numbers (plus does a validated tool exist I'm not sure). I think GPs much prefer to look at the responses in a holistic sense rather than the total "score". Scores are not holistic." (Disagreeing GP)

F Data collecting and collating should be streamlined to avoid duplication of work in clinical tasks and in quality improvement activities

i. The calculated antenatal screening scores should be encoded in such a way that they auto-populate relevant fields in health summaries, electronic referrals and shared maternity care records, with patient consent.

ii. Data on antenatal screening tool use and outcomes should be readily accessible to clinicians to support quality improvement activities, and to assist them in demonstrating key performance indicators to funders.

Sense test results, N=22

Strongly agree	Agree	Neutral	Disagree	Strongly disagree
**10**	6	4	2	
1 x professional association / College representative		2 x GP, 1 x vendor, 1 x other primary clinician	1 x vendor, 1 x GP	

Comments from sense testing respondents: *"Yes, doctors are busy enough" (Strongly agreeing GP)*
"If it isn't automated, it won't happen" (Strongly agreeing GP)
"I like the auto population but not the score, but the actual data about alcohol drugs etc." (Neutral GP)
"Point (i) I would say this is an optional data field." (Neutral vendor)
"Not sure whether there is much benefit in it." (Disagreeing vendor)

Development process recommendations

G Clinical decision support tools should be co-designed with end users

i. Digital antenatal screening tools (with reference to Recommendation A) should be co-designed with clinicians working in a range of clinical settings to ensure that they fit well into existing clinical workflows to facilitate greater rates of screening, and collaboratively with primary care clinical software vendors (to ensure technical feasibility) and other experts as appropriate.

ii. Digital antenatal screening tools should be validated as culturally safe and acceptable to a diverse group of patients including people of Aboriginal and Torres Strait Islander and Culturally and Linguistically Diverse backgrounds.

Sense test results, N=22

Strongly agree	Agree	Neutral	Disagree or	Strongly disagree
**16**	6	Nil	Nil	Nil
1 x professional association / College representative	3 x vendors			

Comments from sense testing respondents: *"GPs tend not to be too tech- savvy." (Strongly agreeing GP)*
"Absolutely essential. And it should be multiple users in multiple different workplaces to try to get the balance right (it will most likely be different requirements in Aboriginal Health Centres compared with private GP practice compared with private GP Obstetrician practice)." (Strongly agreeing GP)
"Systems generated outside general practice rarely fit well with Australian GP workflow." (Strongly agreeing GP)
"As long as everyone has some input and a consensus is reached." (Agreeing vendor)

H Ensure appropriate end-user education to encourage uptake

i. Implementation of new antenatal screening tools should be accompanied by clinician education to facilitate uptake using existing channels such as education sessions through software vendors, Primary Health Networks and professional bodies including the Royal Australian College of General Practitioners, Australian College of Rural and Remote Medicine and Australian Primary Health Care Nurses Association.

Sense test results, N=22

Strongly agree	Agree	Neutral	Disagree or	Strongly disagree
**12**	9	1	Nil	Nil
1 x vendor	1 x vendor<br>1 x professional association / College representative	1 x vendor		

Comments from sense testing respondents: *"Agreed - any funding needs to follow to permit effective education" (Strongly agreeing GP)*

"So many times products are implemented without the knowledge of end users." (Strongly agreeing GP)
"Yes too many resources get created but are not implemented successfully. This should be part of the codesign process." (Strongly agreeing GP)
"I think any training should also show clinicians why such a tool is useful and the value it adds to their patient's care (such as the conversations the tool can facilitate and any next steps that are recommended after screening)." (Agreeing GP)

I Consultation, funding, support and clear guidance for primary care clinical software vendors

i. Following from Recommendation G(i), the specifications for the technical design for a new antenatal screening tool for primary care clinical software should be clearly defined, utilise industry standards, and take into consideration the differences in software interface and architecture across different software packages commonly used in relevant primary or community care settings.

ii. For consistent and timely integration of a new antenatal screening tool into multiple primary care clinical software packages, an oversight body should contract with the software developers / vendors and provide them with funding, and make available technical support as required (which could be via consultant project managers).

Sense test results, N=22

Strongly agree	Agree	Neutral	Disagree	Strongly disagree
8	**13**	1	Nil	Nil
1 x vendor	2 x vendors<br>1 x professional association / College representative	1 x GP		

Comments from sense testing respondents: *"Yes, many good ideas don't get taken up by software developers unless they have good reason to implement them." (Strongly agreeing GP)*
"Yes, integrated assessment not a stand alone tool." (Strongly agreeing GP)

Reimbursement and guideline reform to remove barriers to routine screening

J Reform Medicare Benefits Scheme (MBS) rebates to facilitate antenatal and preconception screening to reflect clinical practice guidelines

i. Appropriate remuneration for antenatal care is an important facilitator of antenatal and preconception screening that aligns with clinical practice guidelines and should reflect the time burden and clinical complexity of screening and enacting management as indicated by screening results.

ii. Remuneration for antenatal screening through the MBS should align with the clinical practice guideline recommendations that screening is conducted as early as possible in a pregnancy, and should be repeated at multiple timepoints in a pregnancy, rather than MBS reimbursement being limited to a single episode after 28 weeks of pregnancy.

Sense test results, N=22

Strongly agree	Agree	Neutral	Disagree	Strongly disagree
**16**	4	2	Nil	
1 x vendor	1 x vendor<br>1 x professional association / College representative	1 x vendor<br>1 x GP		

Comments from sense testing respondents: *"Time is money. GPs already do a lot of work which is not remunerated." (Strongly agreeing GP)*
"Vital. Need to include partners too." (Strongly agreeing GP)
"Yes but I don't like introduction of new item numbers it is too confusing and complex for GPs to keep up." (Strongly agreeing GP)
"The MBS needs to support and reflect the intergenerational importance of antenatal screening, throughout pregnancy. This should not be limited to just formal shared care arrangements." (Strongly agreeing GP)
"I would potentially be concerned about confidentiality if there was a specific antenatal care MBS item - as at times, patients may not want their partners, family members, or other health services to find out that they are pregnant (and this information may be accessible through Medicare if an antenatal item number has been billed). Perhaps this could be done safely, but I think it would need to be carefully considered. Perhaps instead, it would be great if longer consults as are often needed for antenatal care and for discussing complex issues like substance use in pregnancy were remunerated appropriately." (Neutral GP)
